# HERC2 deficiency activates C-RAF/MKK3/p38 signalling pathway altering the cellular response to oxidative stress

**DOI:** 10.1007/s00018-022-04586-7

**Published:** 2022-10-14

**Authors:** Joan Sala-Gaston, Leonardo Pedrazza, Juanma Ramirez, Arturo Martinez-Martinez, Lettie E. Rawlins, Emma L. Baple, Andrew H. Crosby, Ugo Mayor, Francesc Ventura, Jose Luis Rosa

**Affiliations:** 1 Department of Physiological Sciences, Bellvitge Biomedical Research Institute (IDIBELL), University of Barcelona (UB), C/ Feixa Llarga s/n, 08907 L’Hospitalet de Llobregat, Spain; 2 Department of Biochemistry and Molecular Biology, Faculty of Science and Technology, UPV/EHU, Leioa, Bizkaia Spain; 3 RILD Wellcome Wolfson Medical Research Centre, RD&E (Wonford) NHS Foundation Trust, University of Exeter Medical School, Exeter, UK; 4 Peninsula Clinical Genetics Service, Royal Devon & Exeter Hospital (Heavitree), Exeter, UK; 5 Ikerbasque, Basque Foundation for Science, 48013 Bilbao, Spain

**Keywords:** Neurodevelopmental disorder, Angelman, Ubiquitin, MAPK, Cell stress

## Abstract

*HERC2* gene encodes an E3 ubiquitin ligase involved in several cellular processes by regulating the ubiquitylation of different protein substrates. Biallelic pathogenic sequence variants in the *HERC2* gene are associated with HERC2 Angelman-like syndrome. In pathogenic *HERC2* variants, complete absence or marked reduction in HERC2 protein levels are observed. The most common pathological variant, c.1781C > T (p.Pro594Leu), encodes an unstable HERC2 protein. A better understanding of how pathologic *HERC2* variants affect intracellular signalling may aid definition of potential new therapies for these disorders. For this purpose, we studied patient-derived cells with the HERC2 Pro594Leu variant. We observed alteration of mitogen-activated protein kinase signalling pathways, reflected by increased levels of C-RAF protein and p38 phosphorylation. HERC2 knockdown experiments reproduced the same effects in other human and mouse cells. Moreover, we demonstrated that HERC2 and RAF proteins form molecular complexes, pull-down and proteomic experiments showed that HERC2 regulates C-RAF ubiquitylation and we found out that the p38 activation due to HERC2 depletion occurs in a RAF/MKK3-dependent manner. The displayed cellular response was that patient-derived and other human cells with HERC2 deficiency showed higher resistance to oxidative stress with an increase in the master regulator of the antioxidant response NRF2 and its target genes. This resistance was independent of p53 and abolished by RAF or p38 inhibitors. Altogether, these findings identify the activation of C-RAF/MKK3/p38 signalling pathway in HERC2 Angelman-like syndrome and highlight the inhibition of RAF activity as a potential therapeutic option for individuals affected with these rare diseases.

**Supplementary Information:**

The online version contains supplementary material available at 10.1007/s00018-022-04586-7.

## Introduction

Hereditary neurodevelopmental disorders arise from alterations in central nervous system development and manifest perinatally or during infancy and childhood. Despite showing wide genetic and clinical heterogeneity, most share some common phenotypic features, such as developmental delay, impaired motor function and intellectual disability. The identification of genes responsible for these disorders has enabled genetic diagnosis, accurate genetic counselling, and better management [1].

The *HECT and RCC1-like domain 2 (HERC2)* gene encodes an unusually large protein with 4834 amino acid residues. The HERC2 protein is an E3 ubiquitin ligase that functions in ubiquitylation by accepting ubiquitin from ubiquitin-conjugating enzymes (E2) and transferring it to a target protein [2]. Ubiquitylation affects proteins in many ways, variously marking them for proteasome degradation or, affecting their activity, localisation or interactions with other proteins. Therefore, ubiquitin ligases are key regulators of many cellular processes, with their dysregulation being common in numerous cancers and neurodegenerative diseases [3]. For example, *HERC2* mutations are associated with breast, skin (melanoma), gastric, colorectal, and haematological (leukaemia) cancers [4]. The underlying molecular mechanism could be that HERC2 regulates BRCA1, XPA, USP20 or RPA2 protein ubiquitylation, involved in regulating DNA repair and genomic stability [5–9]. HERC2 also regulates p53 transcriptional program by promoting p53 tetramerisation and subsequent activation, independent of its ubiquitin ligase activity [10–12].

Besides, HERC2 is essential during embryonic development and plays an important role in regulating motor coordination [13]. Moreover, it is highly expressed in the nervous system and has been linked with hereditary neurodegenerative disorders [14]. Biallelic *HERC2* variants associated with HERC2 Angelman-like syndrome include missense and frameshift mutations with a premature stop codon that result in a loss of function. These cases are associated with a complete loss or markedly reduced levels of HERC2 protein [15–19]. The condition was first described in Amish/Mennonite communities, associated with homozygosity for a *HERC2* (c.1781C > T, p.Pro594Leu) founder gene variant at increased frequency in the population (autosomal recessive mental retardation type 38; OMIM # 615516) [15, 16]. Proteomic studies of peripheral blood-derived lymphoblasts from individuals with this condition suggest derangements of multiple cellular pathways probably involving disparate pathogenic mechanisms [20]. Despite these efforts, the molecular mechanisms underlying HERC2-related disorders remain elusive, impeding efforts to find potential treatments for these rare diseases. Further investigation of their molecular basis could reveal not only the underlying pathology but also potential therapeutic targets.

In this study, we analysed intracellular signalling pathways in skin fibroblasts from individuals with the pathological variant HERC2 Pro594Leu (HERC2 P594L). They displayed altered mitogen-activated protein kinase (MAPK) signalling that affected the oxidative stress response, with increases in C-RAF protein levels and MAPK p38 activation. These effects were reproduced in other human and mouse cells with HERC2 protein knockdown. Furthermore, we showed that HERC2 regulates C-RAF ubiquitylation and that HERC2 deficiency triggers MKK3/p38 pathway activation in a RAF-dependent manner. In line with this, cells with the HERC2 P594L variant had increased resistance to H_2_O_2_-induced oxidative stress, dependent on the activities of RAF and p38. Finally, we discuss both the implications of these findings for neurodevelopmental disorders caused by HERC2 variants and the potential therapeutic use of RAF inhibitors.

## Materials and methods

### Human cell sample, cell lines and culture conditions

Samples of human skin fibroblasts were obtained with approved informed consent as previously described elsewhere [16].

U2OS, HEK 293T, H1299, RAW 264.7, mouse embryonic fibroblasts (MEFs) and human skin fibroblasts were cultured in Dulbecco’s modified Eagle’s medium supplemented with 10% fetal bovine serum, 2 mM l-glutamine, 100 U/mL penicillin, and 0.1 mg/mL streptomycin sulphate. Mouse primary osteoblasts were cultured in Minimum Essential Medium α with 10% FBS, 2 mM l-glutamine, 1 mM pyruvate, 100 U/ml penicillin, and 0.1 mg/ml streptomycin with 50 μg/ml ascorbic acid and 4 mM β-glycerophosphate. All cells were maintained in a humidified incubator at 37 °C and 5% CO_2_ atmosphere.

### Cell treatments and induction of cellular stress

Cells were treated with one of three inhibitors, as indicated: 1 µM LY3009120 (Selleckchem), 1 µM Sorafenib (Santa Cruz Biotechnology) or 10 µM SB203580 (Selleckchem). Different cellular stress types were induced using different stressors: oxidative stress by 500 µM or 50 µM hydrogen peroxide (H_2_O_2_) (Panreac), depending on the experiment; saline stress by 100 mM NaCl.

### Plasmid and siRNAs transfections

Plasmid transfection was performed using the Lipofectamine LTX method (15338; Invitrogen, Carlsbad, CA, USA), according to the manufacturer’s instructions. Myc-tagged fragments of HERC2 (F1, F2, F3, F4, F5 and F5CT) were kindly provided by Dr. Ohta [21]. Green fluorescent protein (GFP) and C-RAF fusion constructs (CR1, CR2, CR3 and full-length) were generated, sub-cloned and tested elsewhere [22]. Plasmids expressing HERC2 full-length protein pcDNA5 FRT/TO SF-HERC2 (ShB-R) (Addgene plasmid # 55613; http://n2t.net/addgene:55613; RRID:Addgene_55613) and pcDNA5 FRT/TO SF-HERC2 C4762S (ShB-R) (Addgene plasmid # 55614; http://n2t.net/addgene:55614; RRID:Addgene_55614) were a gift from David Chan [23]. His-Ubiquitin constructs were kindly provided by Dr. Erazo [24]. The plasmid expressing a biotinylatable version of ubiquitin had been previously described elsewhere [25].

For gene interference, siRNAs were transfected using the calcium phosphate method described elsewhere [10]. Custom double-stranded siRNA oligonucleotides were obtained from GeneCust (Boynes, France). The forward sequences were as follows: negative control (NC) = 5′-UUCUCCGAACGUGUCACGU**TT**; HERC2 (H2.2) = GACUGUAGCCAGAUUGAAA**TT**; HERC2 (H2.4) = GGAAAGCACUGGAUUCGUU**TT**; HERC1 = CGGCAUGGAUGAACAAAUU**TT**; MKK3 = GGAAGAAGGAUCUACGGAU**TT**; C-RAF = UAGUUCAGCAGUUUGGCUA**TT**; A-RAF = AACAACAUCUUCCUACAUGAG**TT**; B-RAF = AAAGAAUUGGAUCUGGAUCAU**TT**; p53 = GACUCCAGUGGUAAUCUAC**TT**.

### Lentiviral particle production and target cell infection

Lentiviral vectors were produced in HEK 293 T cells. Cells were transfected with 7 μg pMD2.G, 7 μg psPAX2 (VSV-G) and 7 μg of either empty pLKO.1-Puro or pLKO.1‐shHERC2 by the calcium phosphate method. Media containing lentiviral particles were collected, filtered using polyvinyl difluoride filters (Millex-HV filter 0.45 μm, Millipore SLHV033RB) and stored in aliquots at − 80 °C. Target cells were seeded at a confluence of 50–60% in a 6-well plate before adding 300 μL of the medium containing the lentiviral vectors to each well. Fresh medium, supplemented with 5 μg/mL polybrene, was added to make a total volume of 1 mL. Media with lentiviral vectors were removed the next day and after 24 h, 5 μg/mL puromycin was added for selection. MISSION shRNA clone of mouse HERC2 (TRCN0000039444) was purchased from Sigma-Aldrich. The plasmid vector pLKO.1—TRC control was a gift from David Root (Addgene plasmid #10879; http://n2t.net/addgene:10879; RRID:Addgene_10879) [26], and the VSV-G envelope expressing plasmid pMD2.G (Addgene plasmid #12259; http://n2t.net/addgene:12259; RRID:Addgene_12259) and the lentivirus packaging plasmid psPAX2 (Addgene plasmid #12260; http://n2t.net/addgene:12260; RRID:Addgene_12260) were a gift from Didier Trono.

### Protein extraction, PAGE, and immunoblotting

For protein extraction, cells were washed twice in ice-cold phosphate-buffer saline after discarding the media. Cell lysis was performed by scrapping after adding of NP40 lysis buffer (50 mM Tris–HCl, pH 7.5, 150 mM NaCl, 50 mM NaF, 0.5% NP40) containing protease and phosphatase inhibitors as previously described [27]. Lysates were maintained on ice under agitation for 20 min, and then centrifuged at 13,000×*g* at 4 °C for 10 min. Supernatants were collected before analysis using the Tris–Acetate PAGE system [28]. Band intensities were detected using a gel documentation system (LAS-3000, Fujifilm) and quantified with ImageJ software (Rasband, W.S., ImageJ, U. S. National Institutes of Health, Bethesda, Maryland, USA, https://imagej.nih.gov/ij/).

We used the following antibodies: anti-HERC2 monoclonal (BD Biosciences #612366); anti-C-RAF (BD Biosciences #610151); anti-Clathrin Heavy Chain (TD.1) (Santa Cruz Biotechnology #sc12734); anti-P-ERK1/2 (Sigma-Aldrich #M 8159); anti-p44/42 MAPK (ERK1/2) (Cell signalling #9102); anti-phospho-p38 (Cell signalling #9211); anti-p38 (Santa Cruz Biotechnology #sc-535); anti-HERC1 (410) [10]; anti-P-MKK3 (Cell signalling #9231); anti-MKK3 (Proteintech #13898–1-AP); anti-A-RAF (A-5) (Santa Cruz #sc-166771); anti-B-RAF (F-7) (Santa Cruz Biotechnology #sc-5284); anti-HERC2 polyclonal (bvg3) [10]; anti-c-myc (clone 9E10) (Roche #1 667 149); anti-GFP (Abcam #ab13970); anti-Flag M2 (Sigma-Aldrich #F 3165); anti-p-HSP27 (Enzo Life Sciences #ADI-SPA-523); anti-HSP27 (Santa Cruz Biotechnology #sc-1049); anti-NRF2 (Cell signalling #12721); anti-ubiquitylated proteins (clone FK2; Biomol); and peroxidase-conjugated secondary antibodies (Invitrogen).

### Confocal microscopy

We seeded U2OS cells on glass coverslips and performed fixation by incubating cells at room temperature for 20 min in 4% paraformaldehyde. Then, cells were permeabilised for 20 min with 0.05% saponin in phosphate-buffered saline containing 0.5% bovine serum albumin. The primary antibody, anti-phospho-p38 (Cell signalling #9211) (1:200), was incubated at 37 °C for 1 h. After washing, Alexa-Fluor 488 secondary antibody (Invitrogen) (1:500) was incubated at 37 °C for 45 min. Actin filaments were stained by incubation with phalloidin-Alexa 647 (BioProbes) (100 ng/mL) for 20 min at room temperature. Nuclei were stained with DAPI (Sigma-Aldrich) (1 μg/mL). All images were acquired using a confocal laser scanning microscope (LSM 880 spectral, Carl Zeiss Microscopy GmbH, Jena, Germany).

### Immunoprecipitation and pull-downs

For immunoprecipitation, cells were lysed with CHAPS buffer (10 mM Tris–HCl, pH 7.5, 100 mM NaCl, 0.3% CHAPS) containing protease and phosphatase inhibitors as described above. Cell lysates (input) were incubated with pre-immune serum or anti-HERC2 polyclonal antibody (bvg3) for 2 h at 4 °C with gentle rotation and immunoprecipitated with protein A-Sepharose (GE Healthcare) for 1 h at 4 °C. Beads were pelleted by centrifugation at 2500×*g*, washed five times with CHAPS buffer, and analysed by electrophoresis and immunoblot.

For the GFP pull-downs, supernatants were incubated with 2 μL GFP-TrapA (ChromoTek) for 2 h at 4 °C. Pellets were washed five times with CHAPS buffer and analysed by electrophoresis and immunoblot.

For ubiquitome proteomic experiments, biotin-pull-downs were performed in triplicates as previously described [25], in order to compare proteins more ubiquitinated in Flag-HERC2 WT-overexpressing cells, relative to Flag-HERC2 C4762S-overexpressing cells.

### Ubiquitylation assay

HEK 293 T cells were transfected with the indicated plasmids for 48 h. Before the ubiquitylation assay, the cells were treated for 4 h with 10 µM of the proteasome inhibitor MG132 (Sigma-Aldrich/Merck #C2211). Then, cells were lysed with denaturing buffer #1 (6 M guanidinium-HCl, 10 mM Tris, 100 mM Na_2_HPO_4_–NaH_2_PO_4_ buffer, pH 8) and the cells extracts were incubated with the nickel beads (Ni^2+^-NTA agarose; Qiagen) for 2 h at 4 °C under rotation. Beads were successively washed as follows: twice with 1 ml of denaturing buffer #1 plus 10 mM 2-mercaptoethanol; three times with 1 ml of buffer #2 (8 M urea, 10 mM Tris, 10 mM 2-mercaptoethanol, 100 mM Na_2_HPO_4_–NaH_2_PO_4_ buffer, pH 8); twice with 1 ml of buffer #3 (8 M urea, 10 mM Tris, 100 mM Na_2_HPO_4_–NaH_2_PO_4_ buffer, pH 6.3) containing 0.2% Triton X-100; once with 1 ml of buffer #3 containing 0.1% Triton X-100 and 0.5 M NaCl; and three times with 1 ml of buffer #3. Finally, proteins were eluted by incubating the beads with 200 mM imidazole in 5% SDS, 0.15 M Tris–HCl, pH 6.7, 30% (vol/vol) glycerol, 0.72 M 2-mercaptoethanol for 1 h at 37 °C with mixing. The samples were analysed by immunoblotting as indicated above.

### Reverse transcription and quantitative PCR

Total RNA was isolated from U2OS cells using the TRIsure reagent according to the manufacturer’s protocol (Bioline). Total RNA (2 μg) was reverse-transcribed using the high-capacity cDNA Reverse Transcription kit (Applied Biosystems). PCR amplification reactions were performed with the ABI Prism 7900 HT Fast Real-Time PCR System. Applied Biosystems’ TaqMan Gene Expression Assays (ThermoFisher Scientific) were used to quantify the gene expressions of the following: *GUSB* (Hs00939627_m1), *NFE2L2* (Hs00975960_m1), *SOD1* (Hs00533490_m1), *SOD2* (Hs00167309_m1), *GPX1* (Hs00829989_Gh), and the housekeeping gene *GAPDH* (Hs99999905_m1), which was used to normalise.

### MTT assay for cell viability and cell proliferation

Using 96-well plates, U2OS cells and human skin fibroblasts were seeded to final concentration of 10,000 cells/well or 15,000 cells/well, respectively. After incubation at 37 °C for 24 h in the cell incubator, we initiated treatments, as indicated and performed the MTT assay (M5655; Sigma/Merck) according to manufacturer’s instructions. Briefly, we added MTT at a final concentration of 0.5 mg/mL to each well, incubated the cells for 4 h in a humidified incubator, then discarded the media and solubilised the formazan crystals with isopropanol. Finally, absorbance at a wavelength of 570 nm was determined using a 96-well plate spectrophotometer.

### MitoSox staining

To evaluate mitochondrial reactive oxygen species (ROS), human skin fibroblasts were seeded in a µ-Slide 8 well-chambered coverslip at a concentration of 15,000 cells/well. The next day, cells were stained with 1 µg/mL of Hoechst 33,342 (H3570, ThermoFisher, USA) for 30 min at 37 °C and with 2 µM MitoSOX Red (Invitrogen) for 15 min at 37 °C. Cells were examined in a Zeiss LSM 880 laser scanning confocal spectral microscope equipped with an incubation control system (37 °C, 5% CO_2_). Fluorescence intensity per cell was measured, quantified and expressed as arbitrary units (a.u). Images were analysed using ImageJ software (Rasband, W.S., ImageJ, U.S. National Institutes of Health, Bethesda, Maryland, USA, https://imagej.nih.gov/ij/).

### Mitotracker staining

For mitochondria staining, human skin fibroblasts were seeded in a µ-Slide 8 well-chambered coverslip at a concentration of 15,000 cells/well. The next day, cells were stained with 1 µg/mL of Hoechst 33,342 (H3570, ThermoFisher, USA) and 50 nM Mitotracker Red CMXRos (M7512, ThermoFisher, USA) for 30 min at 37 °C. Images were taken using a Zeiss LSM 880 laser scanning confocal spectral microscope equipped with an incubation control system (37 °C, 5% CO_2_). Fragmented mitochondrial percentage was calculated by counting spherical non-contiguous mitochondrial particles and dividing by the number of total structures comprised in the mitochondrial network. Images were analysed using ImageJ software (Rasband, W.S., ImageJ, U.S. National Institutes of Health, Bethesda, Maryland, USA, https://imagej.nih.gov/ij/).

### Statistical analysis

The results indicate the means and standard error of the mean (± SEM) of, at least, three independent experiments. Individual data points are plotted as single dots. Significance was calculated by Student t-test and indicated as follows: *, **, or *** for *p* values of ≤ 0.05, ≤ 0.01, or ≤ 0.001, respectively. Figures were created, and statistical analysis was performed, using GraphPad Prism version 8.4.3 for Windows (GraphPad Software, San Diego, California USA), www.graphpad.com.

## Results

### Human HERC2 Pro594Leu cells display MAPK pathway alteration

**Fig. 1:**
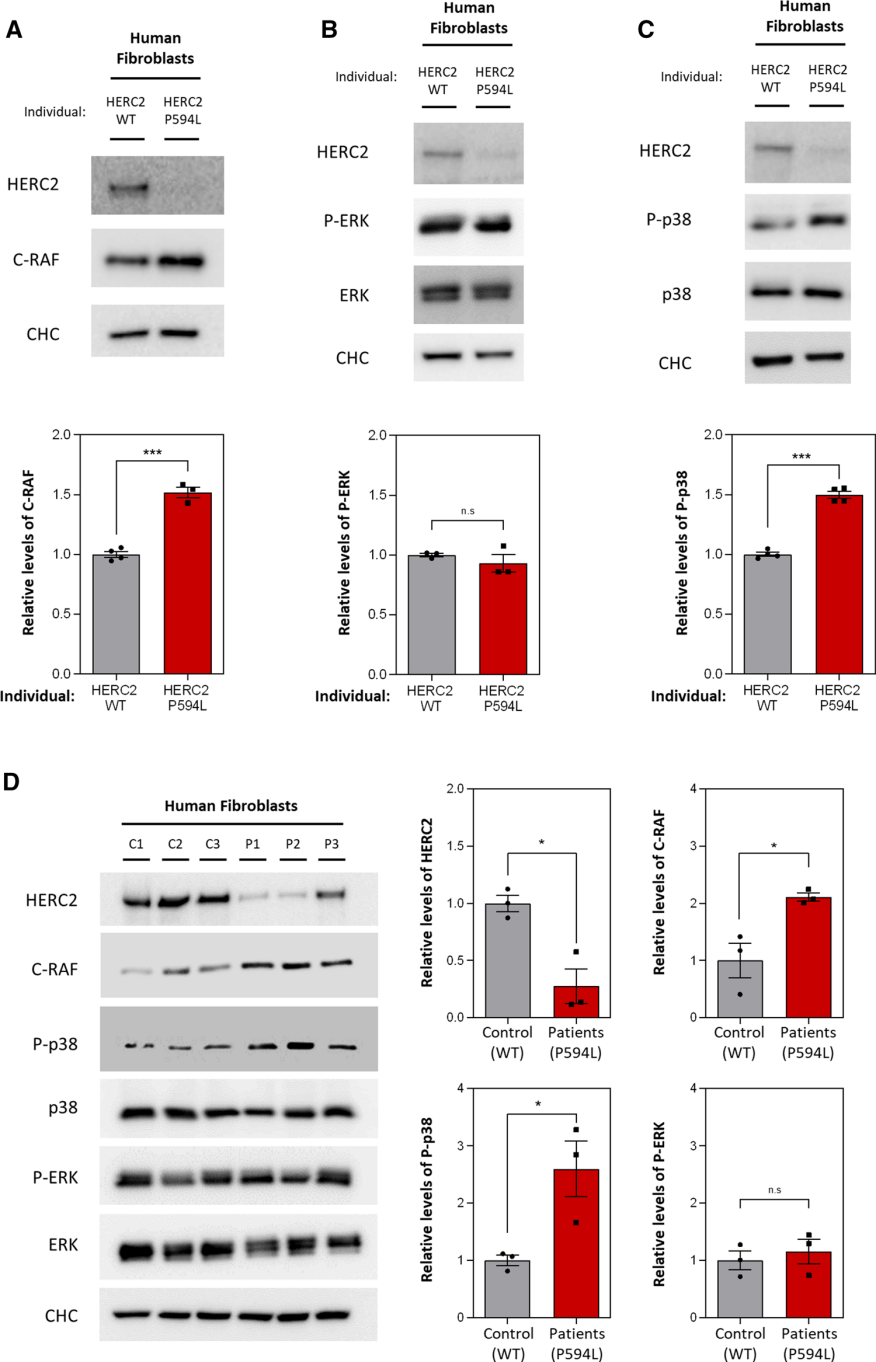
Patient-derived cells with a homozygous mutation in human *HERC2* gene show MAPK pathway alterations. **A–C** We analysed lysates of human skin fibroblasts from an individual with the wild-type HERC2 (HERC2 WT) and the p.Pro594Leu mutant HERC2 variant (HERC2 P594L) by immunoblot, using the indicated antibodies. C-RAF (**A**), phospho-ERK (P-ERK) (**B**) or phospho-p38 (P-p38) (**C**) levels were quantified and normalised based on clathrin heavy chain (CHC), ERK or p38 protein levels, respectively. The results are expressed relative to the control condition. Plots represent mean ± standard error of the mean. Representative results are shown for experiments repeated at least three times and the data points of each experimental repetition are plotted as single dots. (**D**) We analysed lysates of human skin fibroblasts from three different control individuals with the wild-type HERC2 (C1, C2 and C3) and three different patients with the HERC2 P594L mutant variant (P1, P2 and P3) by immunoblot. Levels of HERC2 and C-RAF proteins were quantified as in **A**. Levels of P-ERK and P-p38 were quantified as in **B**, **C**, respectively. The results are expressed relative to the control condition. Plots represent mean ± standard error of the mean. Representative results are shown for experiments repeated at least three times and the data points of each of the individuals analysed are plotted as single dots. Significance levels: ns = non-significance; *p ≤ 0.05; **p ≤ 0.01; ***p ≤ 0.001

Several recessive mutations affecting the *HERC2* gene cause developmental delay with Angelman-like features [14, 19]. Knowing how pathologic *HERC2* variants affect intracellular signalling could reveal the underlying pathology and identify possible therapies. Therefore, we studied cells from an individual with the mutant HERC2 P594L variant described in most cases. Since HERC1 had previously been reported to regulate the ERK and p38 MAPK signalling pathways [22, 29], we wondered if HERC2 also had a modulatory role. As expected, cells with the HERC2 P594L mutation showed almost undetectable HERC2 protein levels (Fig. 1A–C). Interestingly, although they showed higher protein levels of C-RAF (Fig. 1A), this did not correlate with the canonical activation of the ERK signalling pathway, assessed by ERK phosphorylation (Fig. 1B). An increment in p38 phosphorylation was also detected while total p38 protein levels remained stable (Fig. 1C).

In order to provide more evidence that these changes in MAPK signalling pathways are a general hallmark of disease in patients with biallelic HERC2 mutations, we analysed samples of two more individuals carrying the mutant HERC2 P594L variant. Consistently, patients with the HERC2 P594L mutation (P1, P2 and P3) showed lower HERC2 protein levels than the wild-type controls (C1, C2 and C3). In addition, C-RAF protein levels and p38 phosphorylation were upregulated in all three patients, but no changes were detected in ERK activation (Fig. 1D). These results showed that cells with the HERC2 P594L mutation exhibit altered MAPK signalling pathway activation, as reflected by higher C-RAF and phospho-p38 protein levels.

### HERC2 regulates C-RAF protein levels

**Fig. 2:**
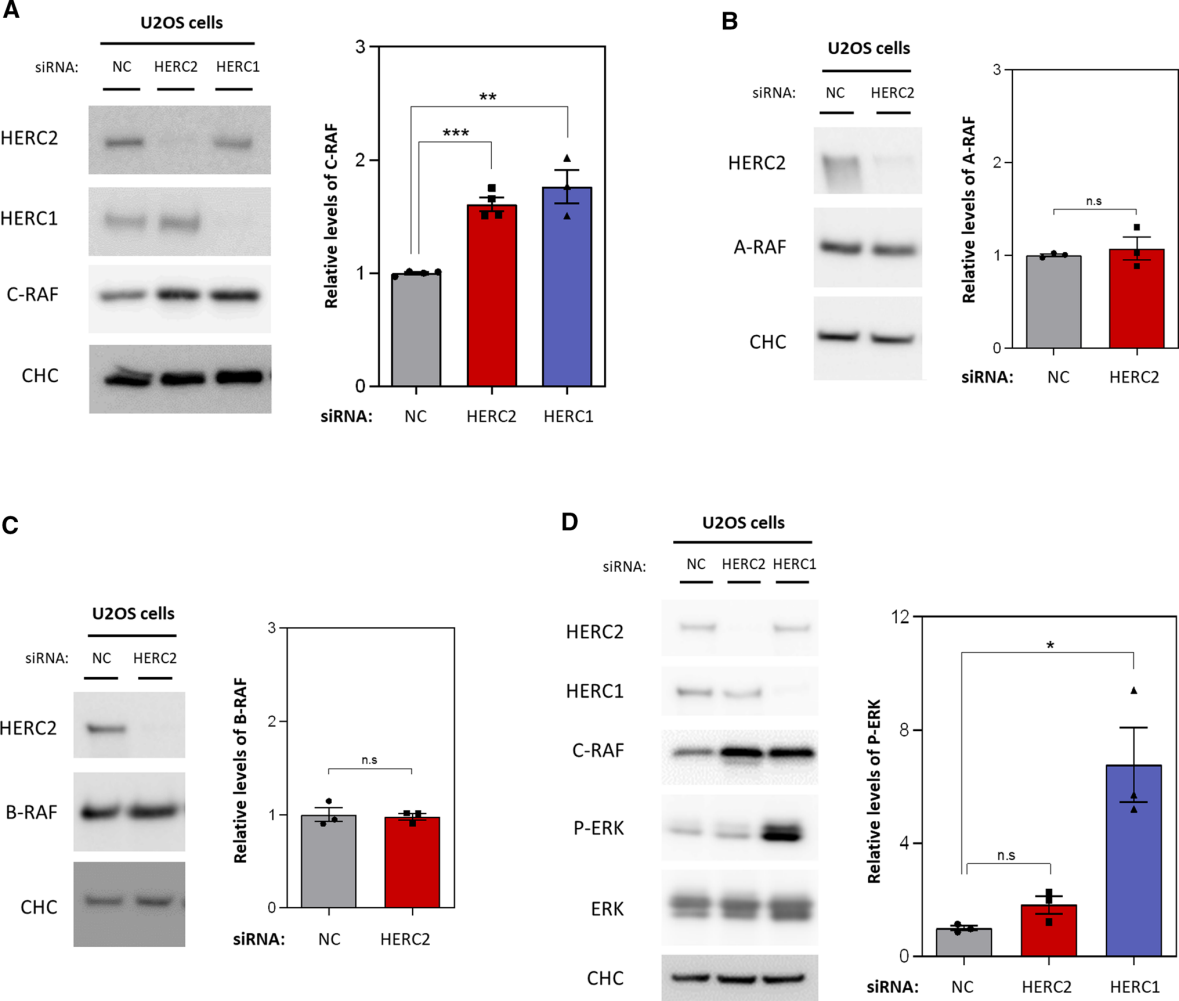
HERC2 regulates C-RAF protein levels. **A** U2OS cells were transfected with an siRNA negative control (NC), an siRNA against HERC2, or an siRNA against HERC1. The indicated protein levels were analysed by immunoblot. Levels of C-RAF proteins were quantified, normalised based on clathrin heavy chain (CHC) protein levels (loading control), and expressed relative to the control condition. **B**, **C** U2OS cells were transfected with a NC siRNA or an siRNA against HERC2. Levels of A-RAF (**B**) or B-RAF (**C**) were analysed by immunoblot, quantified, normalised based on CHC protein levels, and expressed relative to the control condition. (**D**) U2OS cells were transfected as in **A**, and the indicated protein levels were analysed by immunoblot. Phospho-ERK (P-ERK) levels were quantified, normalised based on ERK protein levels and expressed relative to the control condition. Plots represent the mean ± standard error of the mean. Representative results are shown from experiments repeated at least three times and the individual data points are plotted as single dots. Significance levels: ns = non-significance; *p ≤ 0.05; **p ≤ 0.01; ***p ≤ 0.001

To delve deeper into the molecular mechanisms involved in the altered MAPK signalling pathway in HERC2 P594L cells, we considered human cells with low levels of HERC2 protein shared this alteration. In knockdown experiments performed in human U2OS cells, cells were transfected with either a negative control (NC) small-interfering RNA (siRNA), an siRNA against HERC2, or a positive control siRNA against HERC1. The positive control was chosen because previous work had shown that HERC1 controls ERK and p38 signalling pathways modulating C-RAF protein levels [22, 29]. HERC2 knockdown mimicked the effect observed in HERC2 P594L cells, with depletion of HERC2 correlating with increased C-RAF protein levels. As expected, this was also observed after HERC1 silencing (Fig. 2A). HERC2 depletion modified neither A-RAF nor B-RAF protein levels (Fig. 2B, C). These data indicated that RAF regulation by HERC2 is specific for the C-RAF isoform.

Next, we analysed the RAF MAPK signalling pathway, in which canonical RAF activation triggers ERK phosphorylation [29]. We noted that C-RAF upregulation observed after HERC1 depletion correlated with increased phosphorylated ERK levels, while total ERK protein levels remained stable. However, we detected no changes in ERK phosphorylation in the HERC2-depleted cells (Fig. 2D). These results suggested that C-RAF upregulation caused by HERC2 depletion was not signalled through the canonical MEK/ERK pathway.

### HERC2 regulates p38 phosphorylation

**Fig. 3:**
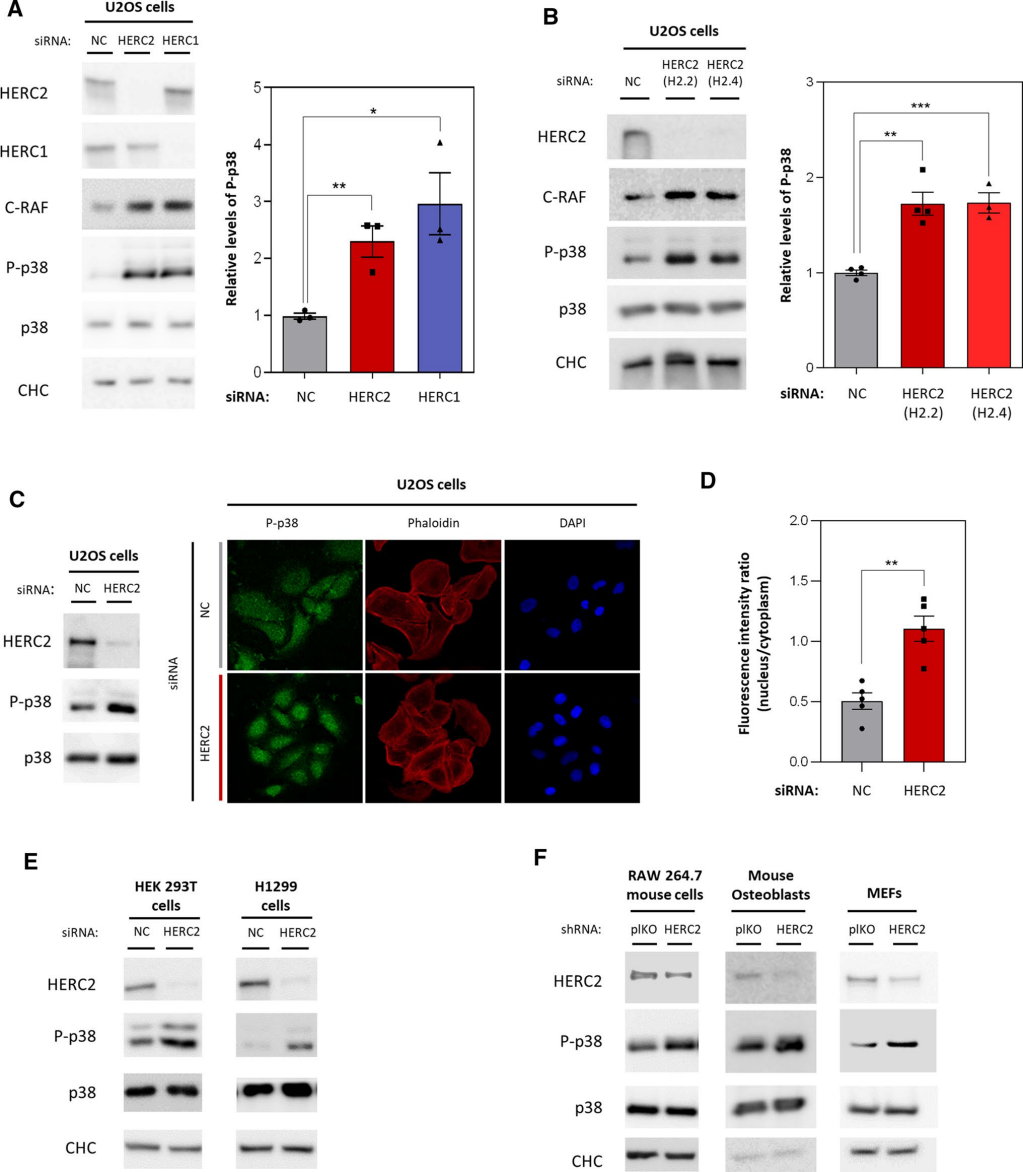
HERC2 regulates p38 phosphorylation. **A** U2OS cells were transfected with an siRNA negative control (NC), an siRNA against HERC2, or an siRNA against HERC1. The indicated protein levels were analysed by immunoblot. Levels of phospho-p38 (P-p38) were quantified, normalised based on total p38 protein levels, and expressed relative to the control condition. **B** U2OS cells were transfected with an siRNA negative control (NC) and two different siRNA sequences against HERC2: H2.2 or H2.4. The indicated protein levels were analysed by immunoblot and phospho-p38 levels were quantified and represented as in (A). **C** U2OS cells transfected with NC or HERC2 siRNA were analysed by immunoblot against the indicated proteins and by confocal microscopy. Fixed cells were stained for phospho-p38 (green), F-actin with phalloidin (red), and nuclei with DAPI (blue) and analysed by immunofluorescence. **D** Fluorescence intensity in the nucleus and cytoplasm per cell was measured and quantified. The ratio nucleus/cytoplasm was calculated. Each data point represent mean of a different field. **E** HEK 293T and H1299 cells were transfected with a NC or HERC2 siRNA. The indicated protein levels were analysed by immunoblot. **F** A RAW 264.7 mouse macrophage cell line, mouse primary osteoblasts and mouse embryonic fibroblasts (MEFs) were infected with lentiviral particles carrying either the empty plKO vector as a negative control (plKO) or an shRNA against HERC2. The indicated protein levels were analysed by immunoblot. Plots represent mean ± standard error of the mean. Representative results are shown from experiments repeated at least three times and the individual data points are plotted as single dots. Significance levels: ns = non-significance; *p ≤ 0.05; **p ≤ 0.01; ***p ≤ 0.001

Given that HERC1 regulates the MKK3/p38 axis through a RAF-dependent mechanism [29], we decided to study if this mechanism was the same for HERC2. We analysed levels of p38 phosphorylation in U2OS cells transfected with a negative control siRNA, an siRNA against HERC2, and a positive control siRNA against HERC1. We observed the induction of p38 phosphorylation in HERC2-depleted cells, though with total p38 protein levels remaining stable and higher C-RAF protein levels (Fig. 3A). Analogous behaviour was detected in HERC1-depleted cells (Fig. 3A). The same results for p38 phosphorylation were obtained when silencing HERC2 with siRNAs containing different RNA sequences (HERC2 H2.2 and HERC2 H2.4) (Fig. 3B).

The phosphorylation of p38 is associated with its activation and nuclear translocation. To check this, we analysed p38 subcellular localisation. Immunofluorescence experiments showed increased p38 nuclear localisation in HERC2-depleted cells (Fig. 3C). This was quantified assessing the nucleus:cytoplasm ratio, which was higher in HERC2-depleted cells compared with control cells (Fig. 3D).

After HERC2 silencing, p38 activation, was replicated in other human cells, such as the p53-lacking human non-small lung carcinoma cell line (H1299) and the non-tumorigenic human kidney 293 T cell line (HEK 293 T) (Fig. 3E). In addition, the same results were obtained in mouse cells and when using a different HERC2 silencing method. RAW 264.7 macrophage cell line, primary mouse osteoblasts and MEFs were infected with lentiviral particles carrying either an empty vector as a control (plKO) or a short hairpin RNA (shRNA) against HERC2. All HERC2 knockdown cells presented higher phospho-p38 protein levels compared to controls, while total p38 protein levels remained constant (Fig. 3F). In conjunction, these results demonstrated that HERC2 participates in regulating p38 signalling.

### HERC2 regulates the MKK3/p38 pathway through crosstalk mediated by C-RAF

**Fig. 4:**
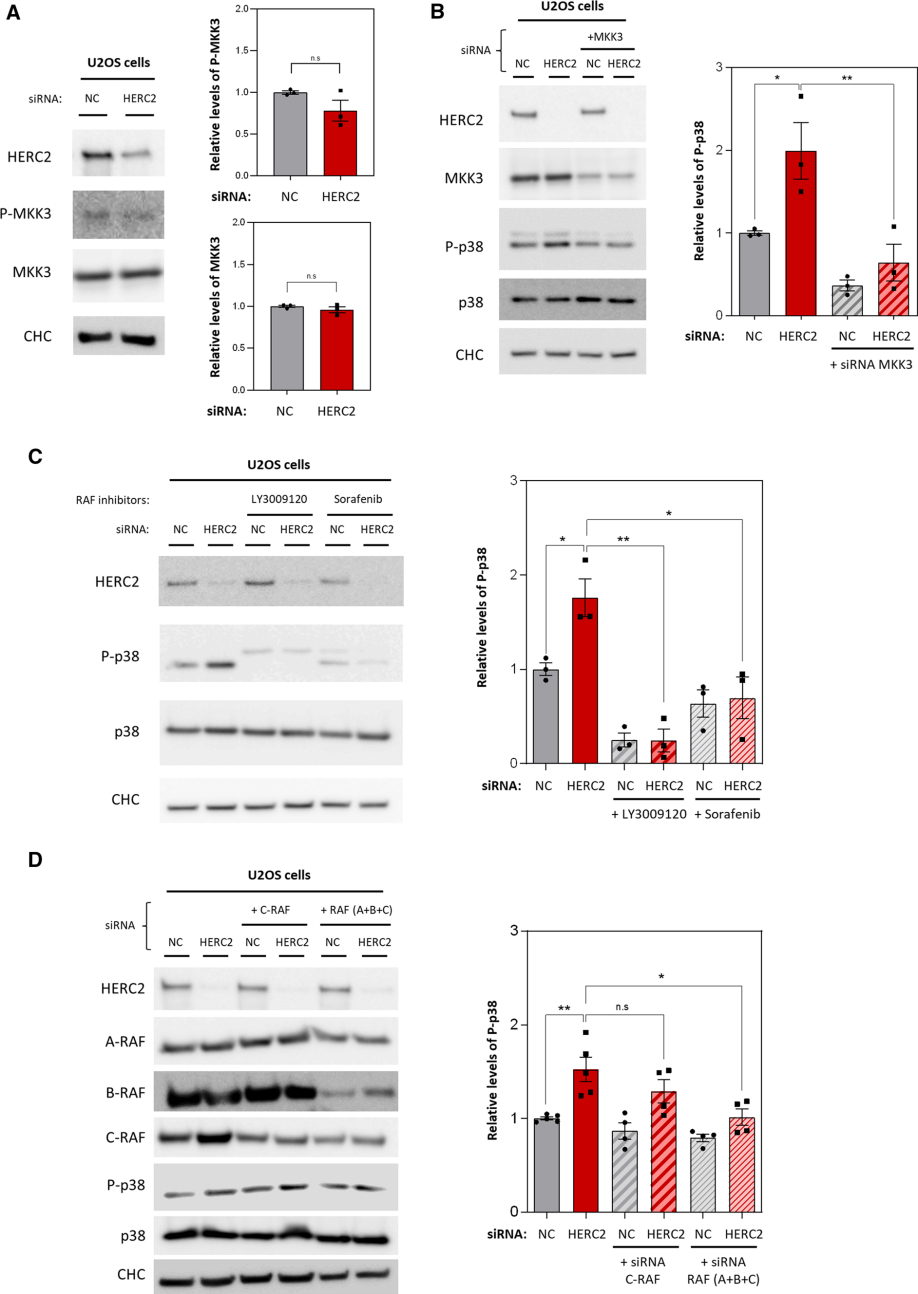
HERC2 regulates the MKK3/p38 pathway through crosstalk mediated by C-RAF. **A** U2OS cells were transfected with an siRNA negative control (NC) or an siRNA against HERC2. The indicated protein levels were analysed by immunoblot. Levels of MKK3 and P-MKK3 were quantified and normalised based on clathrin heavy chain (CHC) protein levels (loading control) and total MKK3 protein levels, respectively, and expressed relative to the control condition. **B** U2OS cells were transfected with the NC or HERC2 siRNA. An siRNA against MKK3 was added in the indicated conditions (+ MKK3) and the indicated protein levels were analysed by immunoblot. Levels of phospho-p38 (P-p38) were quantified, normalised based on total p38 protein levels and expressed relative to the control condition. **C** U2OS cells were transfected with NC or HERC2 siRNA. At 72 h post-transfection, cells were treated with 1 μM of LY3009120 or 1 μM Sorafenib for 1 h. Untreated cells were incubated with dimethyl sulfoxide (DMSO) as a negative control. Lysates were analysed by immunoblotting and phospho-p38 levels were quantified, normalised by total p38 protein levels and expressed relative to the control condition. **D** U2OS cells were transfected with the NC or HERC2 siRNA. C-RAF siRNA alone or a combination of A-, B- and C-RAF siRNAs (A + B + C) was added when indicated. The indicated protein levels were analysed by immunoblot. Phospho-p38 levels were quantified, normalised by total p38 protein levels and expressed relative to control condition. Plots represent mean ± standard error of the man. Representative results are shown from experiments repeated at least three times and the individual data points are plotted as single dots. Significance levels: ns = non-significance; *p ≤ 0.05; **p ≤ 0.01; ***p ≤ 0.001

MAPK kinase (MAPKK or MKK) mediates p38 activation through phosphorylation. MKK3 is the dominant isoform in human U2OS cell lines [29], and its activation has been analysed by measuring its phosphorylation at Ser189 [30]. Thus, we analysed MKK3 activation and its total protein expression in HERC2-depleted U2OS cells, revealing that neither MKK3 phosphorylation at Ser189 nor total MKK3 protein levels were altered compared with control cells (Fig. 4A). To confirm whether p38 phosphorylation triggered by HERC2 depletion depends on MKK3, we co-transfected U2OS with an MKK3 siRNA and either the negative control or the HERC2 siRNA. This revealed that MKK3 knockdown significantly abolished the increment in p38 phosphorylation after HERC2 depletion (Fig. 4B). These data suggested that MKK3 activation caused the increase in phospho-p38 independent of phosphorylation at Ser189.

Given the finding that HERC2 regulates C-RAF and p38 activation, we used two specific RAF kinase inhibitors to identify a potential crosstalk mechanism between the two pathways. LY300912 was used to inhibit all RAF isoforms, and Sorafenib was used to inhibit only B-RAF and C-RAF. In absence of the inhibitors, cells showed an increase in p38 phosphorylation after HERC2 depletion; remarkably, however, this increase was clearly abrogated after incubation with LY3009120 or Sorafenib inhibitors for 1 h (Fig. 4C). Since RAF isoforms interact by forming different heterodimers [31], sometimes all isoforms must be depleted to rescue the regulatory effects mediated by RAF proteins. Therefore, we co-transfected U2OS cells with siRNAs against C-RAF or all three RAF isoforms (A-RAF, B-RAF and C-RAF) along with either the negative control siRNA or the siRNA against HERC2 to achieve knockdown (Fig. 4D). Although silencing C-RAF alone was insufficient to reduce p38 phosphorylation significantly, silencing all three isoforms led to a significant decrease in p38 activation in HERC2-depleted cells (Fig. 4D). Unlike pharmacological inhibition of RAF, triple knockdown failed to produce a complete abrogation of p38 phosphorylation after HERC2 depletion, which is probably due to the fact that siRNA silencing did not achieve sufficient RAF isoforms knockdown. Altogether these results confirm the existence of a crosstalk between the RAF and p38 signalling pathways regulated by HERC2.

### HERC2 interacts with C-RAF

**Fig. 5:**
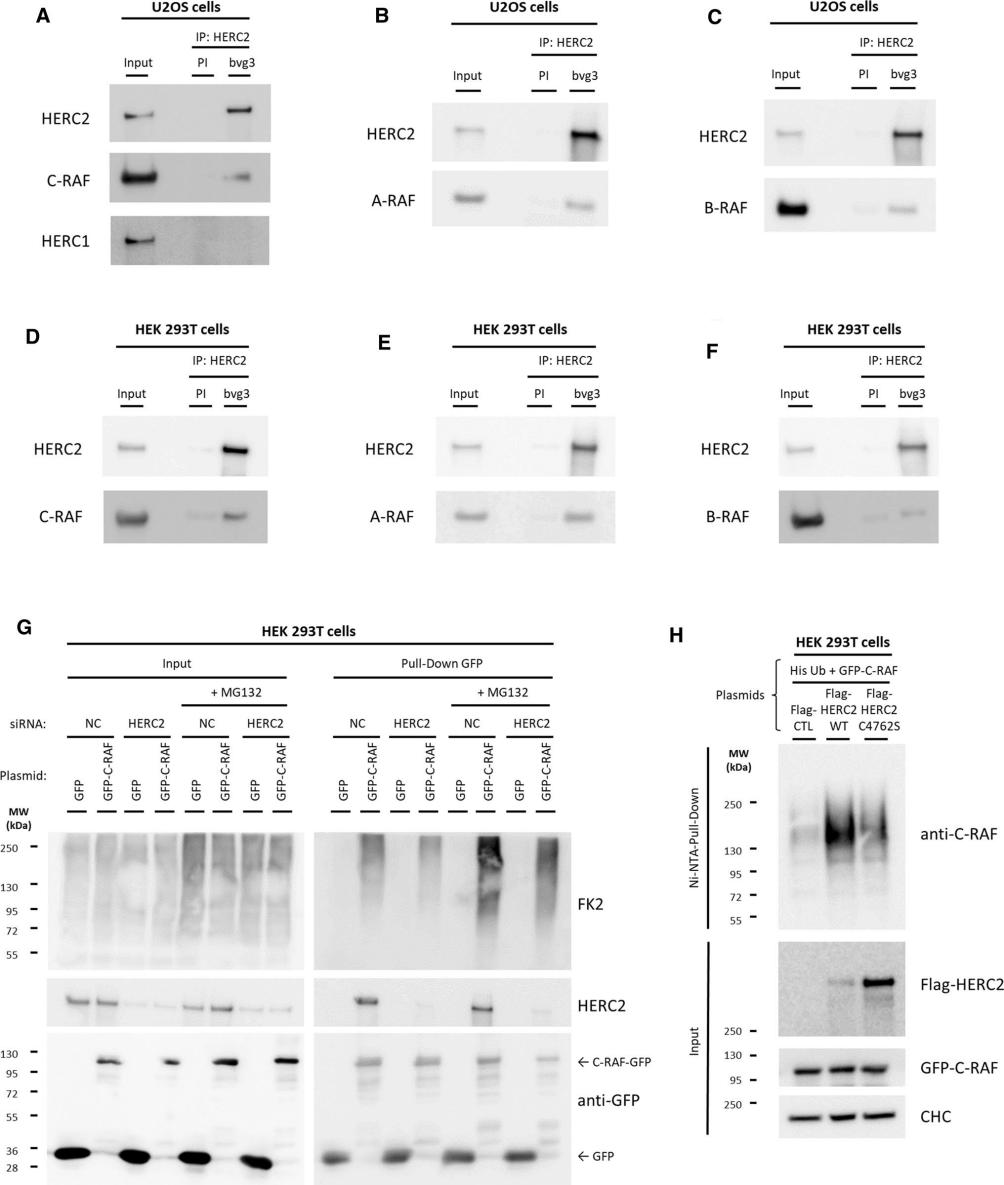
HERC2 interacts with C-RAF and regulates its ubiquitylation. **A**–**F** Supernatants (Input) of lysates from U2OS (**A**–**C**) and HEK 293 T cells (**D**–**F**) were immunoprecipitated (IP) using anti-HERC2 antibodies (bvg3) and analysed by immunoblotting with antibodies against the indicated proteins. Pre-immune serum (PI) was used as a negative control. **G** HEK 293 T cells were transfected with the NC or HERC2 siRNA. Twenty-four hours later, cells were transfected with GFP or GFP-C-RAF plasmids. Forty-eight hours later, cells were incubated for 6 h in the absence or presence of MG132 (10 µM). Lysates were pulled down using GFP resin as indicated in “Materials and methods”. Inputs and proteins retained in the resin (Pull-Down GFP) were analysed by immunoblotting with the indicated antibodies. **H** HEK 293 T cells were transfected with His-Ubiquitin (His-Ub) along with GFP-C-RAF plasmids, as well as a Flag-pcDNA control plasmid (Flag-CTL), an HERC2 WT plasmid (Flag-HERC2 WT), or a catalytically inactive form of HERC2 (Flag-HERC2 C4762S). After 48 h, cells were incubated for 6 h with MG132 (10 µM). Ubiquitylated proteins were purified using a Ni–NTA-agarose resin as indicated in “Materials and methods”. Inputs and pull-downs were analysed by immunoblotting with antibodies against the indicated proteins. Representative results are shown from experiments repeated at least three times

To further investigate the mechanism behind C-RAF regulation by HERC2, we analysed whether these two proteins can interact. In immunoprecipitation experiments in U2OS cells with a specific anti-HERC2 antibody (bvg3), endogenous HERC2 and C-RAF immunoprecipitated, while HERC1 did not, indicating that the interaction of HERC2 and C-RAF was independent of HERC1 (Fig. 5A). RAF hetero-dimerisation between its isoforms is a well-reported process [31], and consistent with this, A-RAF and B-RAF were also detected in HERC2 immunoprecipitated complexes (Fig. 5B, C). The same results were obtained in the human 293 T cell line (Fig. 5D–F).

To identify the region of HERC2 interacting with C-RAF, we co-expressed a GFP-C-RAF fusion protein with a series of Myc-HERC2 fusion proteins in HEK 293 T cells (Supplementary Fig. 1A), followed by pull-down assays with GFP-binding beads. Constructs F4, F5, and F5CT coimmunoprecipitated with GFP-C-RAF, indicating that the HERC2 and C-RAF protein interaction occurs mainly in the carboxyl-terminus of HERC2 polypeptide chain. F5CT construct, which contains the HECT domain holding the ubiquitin ligase activity, showed the highest affinity with C-RAF, suggesting that this is the most relevant interaction site (Supplementary Fig. 1A). HEK 293 T cells were then co-transfected with a Flag-HERC2 full-length fusion protein along with GFP (as a negative control) or the GFP-C-RAF fusion constructs (CR1, CR2, CR3 or full-length) to map the C-RAF region involved. In the GFP pull-down, Flag-HERC2 was coimmunoprecipitated with CR1, CR3, and the full-length constructs (Supplementary Fig. 1B). To characterise this interaction further, we co-expressed the F4 Myc-HERC2 construct with GFP-C-RAF fusion constructs and performed a GFP pull-down, which showed preferential co-immunoprecipitation of the F4 construct with CR3 (Supplementary Fig. 1C). In parallel, the same experiment was done but with the F5CT Myc-HERC2 construct instead of F4, and this revealed co-immunoprecipitation of F5CT with CR1 and CR3 (Supplementary Fig. 1D). In conjunction, pull-down experiments confirmed the interaction between HERC2 and C-RAF, and indicated the possible domains involved. The HERC2 HECT domain, contained in the F5CT construct, showed the highest affinity for C-RAF and its catalytic domain (CR3), suggesting that the HECT and CR3 domains could be the most relevant at the physiological level. Subsequent structural studies should confirm this relevance.

### HERC2 regulates C-RAF ubiquitylation

Having shown that the ubiquitin E3 ligase HERC2 interacts with C-RAF and regulates its protein levels, we wanted to dissect whether HERC2 regulates C-RAF ubiquitylation targeting it to proteasomal degradation. To determine this, we analysed C-RAF ubiquitylation both in control and HERC2-depleted cells in the absence and presence of the proteasome inhibitor MG132. Control and HERC2-depleted HEK 293 T cells were transfected with constructs expressing GFP-C-RAF or GFP as a negative control. Forty-eight hours later, cells were incubated for 6 h in the absence or presence of MG132 (10 µM). Lysates from these cells were pulled down using GFP resin. Inputs and pull-down proteins were analysed by PAGE/SDS and immunoblotted with the anti-ubiquitylated proteins antibody (FK2) or with specific antibodies against the indicated proteins. GFP-C-RAF polyubiquitylation slightly decreased in HERC2-depleted cells compared to control cells under basal conditions (Fig. 5G, lane 10 compared with lane 12). Treatment with MG132 efficiently caused accumulation of polyubiquitylated GFP-C-RAF due to proteasome degradation inhibition in control cells (Fig. 5G, lane 10 compared with lane 14). Remarkably, under MG132 treatment, GFP-C-RAF polyubiquitylation levels were much lower in HERC2-depleted cells (Fig. 5G, lane 14 compared with lane 16). Altogether, these results demonstrated C-RAF proteasome-dependent degradation and its regulation by HERC2.

To confirm the role of HERC2 regulating C-RAF polyubiquitylation we performed an ubiquitylation assay. First, we checked expression of different HERC2 constructs in HEK 293 T cells. We transfected either a negative control plasmid (Flag-CTL), a plasmid encoding wild-type HERC2 protein tagged with Flag peptide (Flag-HERC2 WT), or a plasmid encoding a mutant variant lacking ubiquitin ligase activity (Flag-HERC2 C4762S). HERC2 overexpression occurred in both Flag-HERC2 WT and Flag-HERC2 C4762S transfected cells, being greater with the mutated form (Supplementary Fig. 2A). Next, HEK 293 T cells were also transfected with GFP-C-RAF and His-tagged ubiquitin constructs. Cells were lysed after incubation with the proteasome inhibitor MG132 to enrich ubiquitylated proteins degraded by the proteasome, and incubated with Ni–NTA agarose resin to pull-down His-tagged ubiquitin molecules and the proteins to which they were attached (Fig. 5H). The results showed a smear of high molecular weight GFP-C-RAF indicating much greater C-RAF ubiquitylation with wild-type HERC2 overexpression. The plasmid construct HERC2 C4762S, which is catalytically inactive, was used to determine if the effect on C-RAF ubiquitylation by HERC2 WT overexpression, was dependent on the ubiquitin ligase activity of HERC2. Despite greater overexpression of the mutated form (HERC2 C4762S), this did not correlate with increased C-RAF ubiquitylation (Fig. 5H). Ubiquitin proteomic analysis further supported these results. In short, HEK 293 T cells were transiently transfected with a biotinylatable version of ubiquitin, along with either Flag-HERC2 WT or Flag-HERC2 C4762S plasmids. Ubiquitylated proteome from each condition was then isolated by a biotin-based pull-down approach [25] and analysed by mass spectrometry. C-RAF protein appeared in Flag-HERC2 WT-overexpressing cells, but not in Flag-HERC2 C4762S-overexpressing cells (Supplementary Fig. 2B, C). These results confirmed that HERC2 regulates C-RAF ubiquitylation.

### HERC2 modulates cellular response to H_2_O_2_-induced oxidative stress

**Fig. 6:**
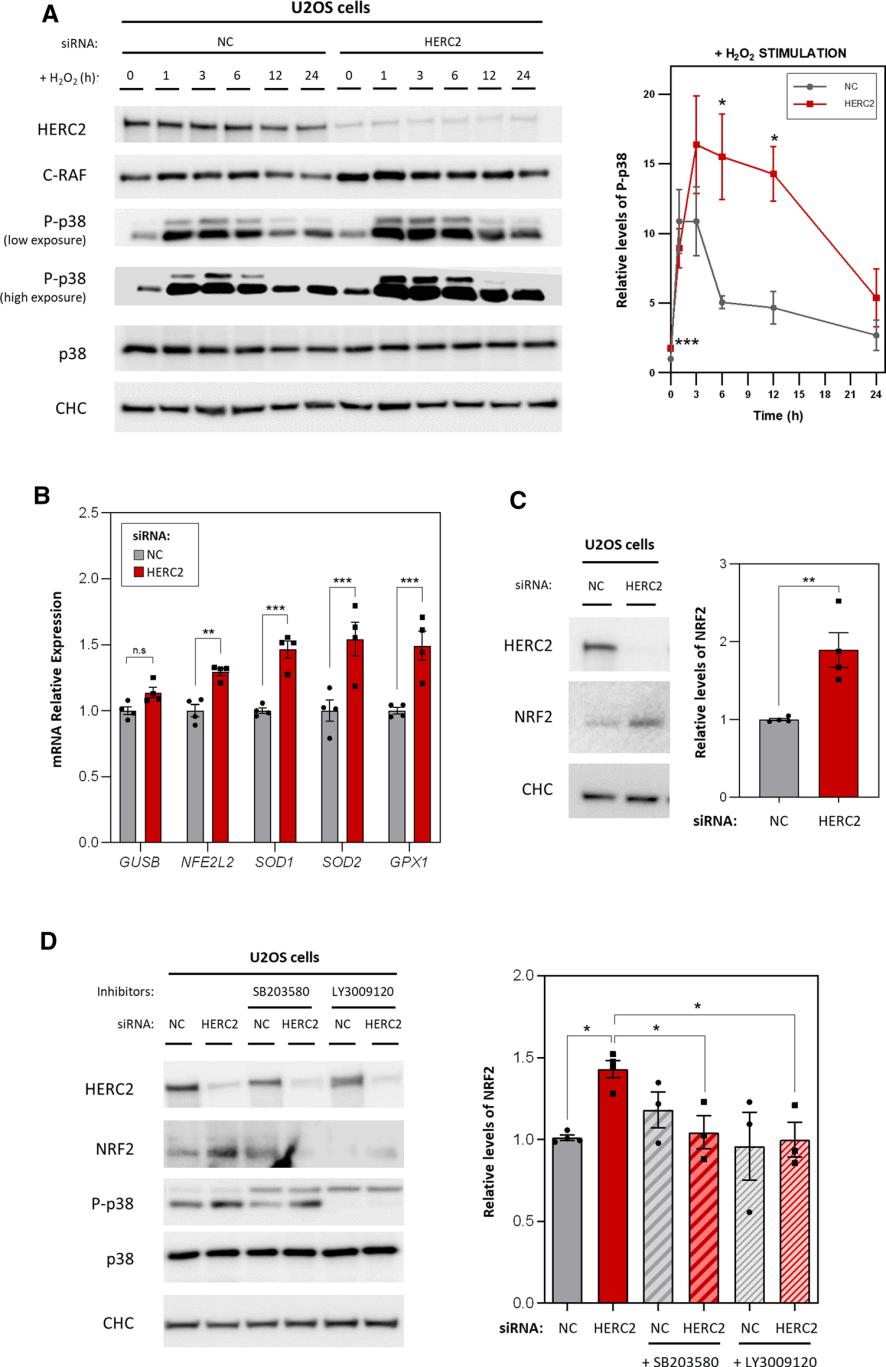
HERC2 modulates cellular response to H_2_O_2_-induced oxidative stress. **A** U2OS cells were transfected with an siRNA negative control (NC) or an siRNA against HERC2. Subsequently, cells were treated with 500 µM H_2_O_2_ to induce oxidative stress for the indicated durations. The indicated protein levels were analysed by immunoblot. Phospho-p38 (P-p38) levels were quantified, normalised based on total p38 protein levels, and expressed relative to the non-treated control condition (NC, *t* = 0). Plots represent mean ± standard error of the mean of 4 independent experiments (*n* = 4). **B** U2OS cells transfected with the NC or HERC2 siRNA were analysed by RT-qPCR. *GUSB*, *NFE2L2*, *SOD1*, *SOD2*, and *GPX1* mRNA expression levels were quantified, and *GAPDH* levels were used to normalise. Each gene quantification is expressed relative to the control condition and individual data points are plotted as single dots. **C** U2OS cells were transfected with the NC or HERC2 siRNA. The indicated protein levels were analysed by immunoblot. NRF2 protein levels were quantified, normalised based on Clathrin Heavy Chain (CHC) protein levels (loading control), and expressed relative to the control condition. **D** U2OS cells were transfected with the NC or HERC2 siRNA. At 72 h post-transfection, cells were treated with 10 µM SB203580 or 1 μM of LY3009120 for 1 h. Untreated cells were incubated with dimethyl sulfoxide (DMSO) as a negative control. Lysates were analysed by immunoblotting and protein levels of NRF2 were quantified, normalised by Clathrin Heavy Chain (CHC) protein levels (loading control), and expressed relative to the control condition. Plots represent mean ± standard error of the mean. Representative results are shown from experiments repeated at least three times and the individual data points are plotted as single dots. Significance levels: ns = non-significance; *p ≤ 0.05; **p ≤ 0.01; ***p ≤ 0.001

Given that a major function of p38 is to regulate cellular stress, we analysed the cellular response to oxidative stress. U2OS cells were transfected with a negative control siRNA or an siRNA against HERC2, and oxidative stress was induced by treating cells with 500 µM H_2_O_2_ for different durations. Protein levels of phosphorylated p38 were analysed by immunoblot. As expected, HERC2-depleted cells started from a more phosphorylated basal state (t = 0) (Fig. 6A). After 3 h of H_2_O_2_ stimulation, both control cells and HERC2-depleted cells clearly showed induced phosphorylation of p38 and reached a maximum peak intensity, which is higher in HERC2-depleted cells. Interestingly, while p38 phosphorylation levels in control cells were clearly reduced after 6 and 12 h of treatment, the HERC2-depleted cells maintained significantly higher levels at these times, resulting in a more pronounced and prolonged phosphorylation response curve (Fig. 6A).

Consistent with HERC2 having a role in regulating the cellular antioxidant response, mRNA levels of the antioxidant genes *NFE2L2*, *SOD1*, *SOD2*, and *GPX1* increased in the HERC2-depleted cells compared with control cells. By contrast, mRNA levels of *GUSB*, used as a negative control, did not change significantly (Fig. 6B). Protein levels of the nuclear factor erythroid 2-related factor 2 (NRF2), a master regulator of all these antioxidants genes, were also upregulated in HERC2-depleted cells (Fig. 6C).

To determine whether the role of HERC2 regulating the cellular response to oxidative stress depends on the activation of the RAF/MKK3/p38 signalling pathway we used p38 (SB203580) and RAF inhibitors (LY3009120). As previously shown, in absence of the inhibitors, cells showed an increase in NRF2 protein levels after HERC2 depletion; however, this increase was abrogated after incubation with SB203580 or LY3009120 for 1 h (Fig. 6D). These results suggested that p38 acts upstream NRF2 activation and that the cellular response to oxidative stress regulated by HERC2 depends on the RAF/MKK3/p38 signalling pathway.

We then evaluated if HERC2 also regulates other stress types modulated by p38. To assess osmotic stress, we treated control cells and HERC2-depleted cells with 100 mM NaCl for different durations; as with H_2_O_2_, the HERC2-depleted cells maintained higher levels of p38 phosphorylation after 6 and 12 h (Supplementary Fig. 3).

Ultimately, these data showed a complex regulation of downstream p38 signalling dependent on HERC2, pointing out HERC2 as a modulator of the cellular response to oxidative and saline stresses.

### HERC2 deficiency alters cellular resistance to H_2_O_2_-induced oxidative stress

**Fig. 7:**
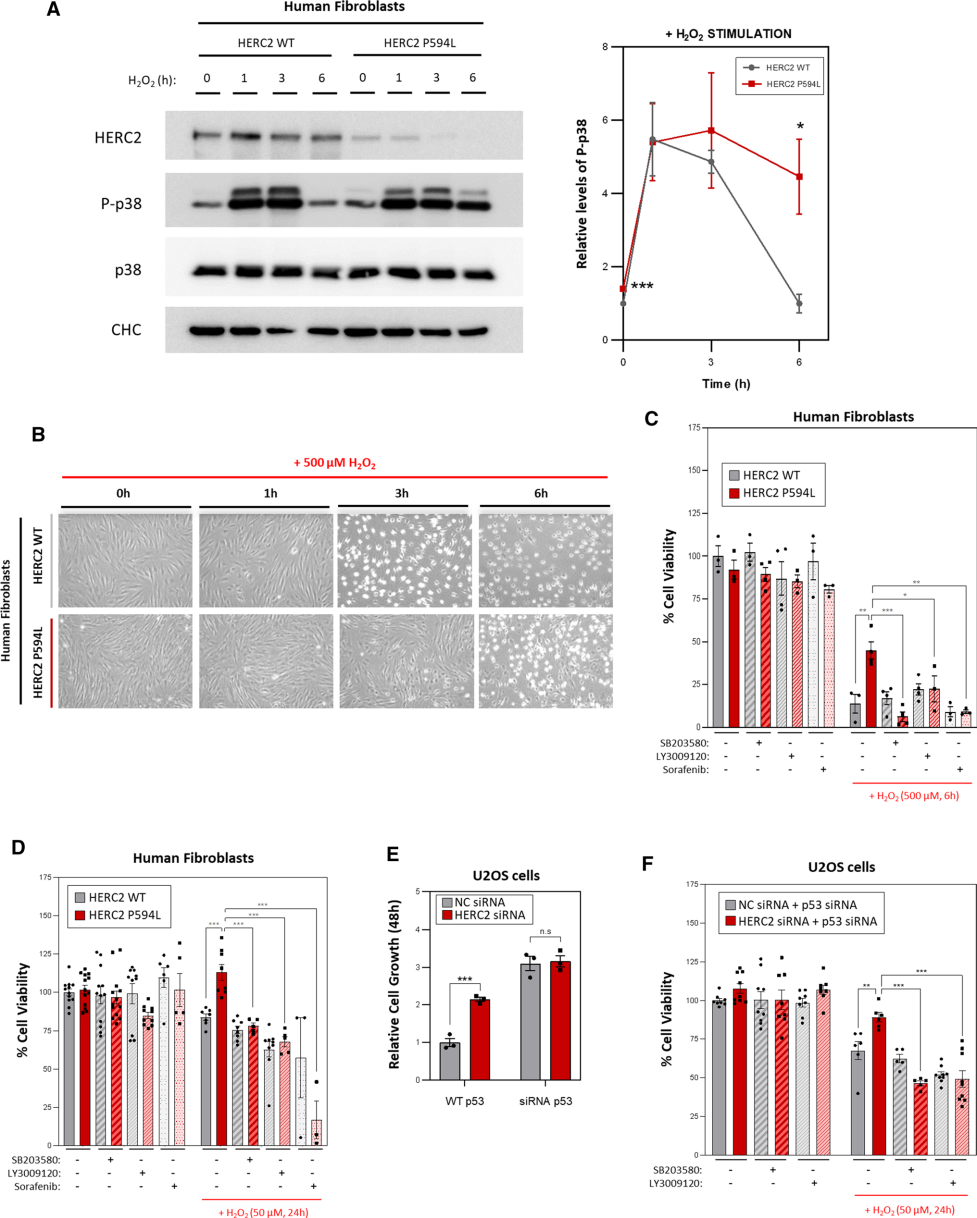
HERC2 deficiency alters cellular resistance to H_2_O_2_-induced oxidative stress. **A** Human skin fibroblasts derived from an HERC2 wild-type individual (HERC2 WT) and an individual with the p.Pro594Leu HERC2 mutant variant (HERC2 P594L) were treated with 500 µM H_2_O_2_ to induce oxidative stress for the indicated time points and protein levels were analysed by immunoblot. Phospho-p38 (P-p38) levels were quantified, normalised based on total p38 protein levels and expressed relative to the non-treated control condition (HERC2 WT, *t* = 0). Plots represent mean ± standard error of the mean of 4 independent experiments (*n* = 4). **B** Human skin fibroblasts were treated as in (A) and images were acquired by optical microscopy after the indicated treatment times, with representative images shown from experiments repeated three times (*n* = 3). **C**, **D** HERC2 WT and HERC2 P594L human skin fibroblasts were treated with 500 µM H_2_O_2_ for 6 h (**C**) or with 50 µM H_2_O_2_ for 24 h (**D**) as indicated. Cells were treated 1 µM LY3009120, 1 µM Sorafenib or 10 µM SB203580 in the specified conditions 1 h before adding H_2_O_2_. An MTT assay was performed. Data are presented as a percentage relative to the control and untreated condition. **E** U2OS were transfected with an siRNA negative control (NC) or an siRNA against HERC2. A p53 siRNA was added when indicated (siRNA p53). Subsequently, cells were plated in a 96-well plate and allowed to grow for 48 h to evaluate cell proliferation and an MTT assay was performed. Data are presented relative to the control condition (NC, WT p53). **F** U2OS cells were transfected with the NC or HERC2 siRNA along with p53 siRNA (NC + p53/HERC2 + p53). Cell viability was assessed by MTT assay (under the same conditions mentioned in **D**). Plots represent mean ± standard error of the mean. Representative results are shown from experiments repeated at least three times and the individual data points are plotted as single dots. Significance levels: ns = non-significance; *p ≤ 0.05; **p ≤ 0.01; ***p ≤ 0.001

Finally, to determine whether cells with the HERC2 P594L mutation showed an altered response to H_2_O_2_-induced oxidative stress, we treated them with 500 µM H_2_O_2_ for different durations. Both the controls (HERC2 WT) and the fibroblasts carrying the mutation (HERC2 P594L) responded with a strong induction of p38 phosphorylation by 1–3 h after H_2_O_2_ treatment. Notably, HERC2 P594L cells maintained higher p38 phosphorylation levels after 6 h, while levels in control cells had already decreased to baseline (Fig. 7A). These differences in p38 signalling correlated with differences in cell morphology spotted by optical microscopy. After 3 h of treatment with H_2_O_2_, the HERC2 WT cells had already begun to show a rounder morphology, probably due to the toxic effect of H_2_O_2_, and after 6 h, most cells showed this altered morphology. By contrast, the HERC2 P594L cells seemed to be more resistant to H_2_O_2_ exposure, appearing healthier and more attached to the plate culture surface than controls at both 3 and 6 h (Fig. 7B). To confirm the differences in cell viability and test their dependence on the C-RAF/MKK3/p38 signalling pathway, MTT assays were performed in the presence of a p38 inhibitor (SB203580) or the RAF inhibitors (LY3009120 or Sorafenib). After 6 h of treatment with 500 µM H_2_O_2_, cell viability fell to 13.7% and 44.8% in the control cells and the HERC2 P594L cells, respectively. The higher resistance of HERC2 P594L cells to H_2_O_2_-induced oxidative stress was abrogated by treatment with the inhibitors (Fig. 7C). We then evaluated this effect under prolonged but less aggressive exposure to H_2_O_2_ (50 µM for 24 h). Again, HERC2 P594L cell viability was higher compared to the controls after stress exposure and, this higher resistance was abrogated by the p38 or RAF inhibitors (Fig. 7D).

Previous studies have reported that HERC2 depletion enhances cell proliferation due to impaired p53 transcriptional activity [10–12]. Given that HERC2 modulates the activity of p53, we wanted to determine whether the evaluated effects on cell viability also depend, in part, on this tumour suppressor protein. As expected, HERC2-depleted cells with functional p53 (WT p53), presented higher cell growth compared to the control cells (Fig. 7E). The differences in cell growth between the control and HERC2-depleted cells were abolished under p53 knockdown (siRNA p53) (Fig. 7E), so we used this model (p53-knockdown U20S cells) to repeat the cell viability assay after H_2_O_2_ exposure. In untreated conditions, no significant differences were observed between negative control cells (NC + p53) and HERC2-depleted cells (HERC2 + p53) (Fig. 7F), including after treatment with the inhibitors. However, after 24 h of treatment with 50 µM H_2_O_2_, cell viability reduced to 67.5% in control cells and only to 89.2% in HERC2-depleted cells. Again, the higher cell resistance of HERC2-depleted cells was abrogated by treatment with p38 (SB203580) and RAF (LY3009120) inhibitors (Fig. 7F). Taken together, these results demonstrated that cellular resistance to H_2_O_2_-induced oxidative stress acquired by HERC2 deficiency is independent of p53, instead being mediated through the C-RAF/MKK3/p38 signalling pathway.

These above results suggested that cells with HERC2 deficiency are better equipped against oxidative stress, so we wondered how does increased protection against oxidative stress fits into pathology. Excessive reactive oxygen species (ROS) cause oxidative stress. However, ROS also play a physiological role in cell signalling. Thus, appropriate ROS production is essential to maintain redox balance. Overexpression of antioxidant enzymes, such as NRF2, may lead the cell to a more reduced state. This pathophysiological situation is known as reductive stress and can be as harmful as is oxidative stress [32–34]. To assess this, mitochondrial ROS levels were evaluated with MitoSOX staining. Cells with the HERC2 P594L mutation showed lesser production of mitochondrial ROS than control cells, suggesting a more reduced state in these cells (Supplementary Fig. 4A). In addition, mitochondria were stained using MitoTracker probes. HERC2 P594L cells presented a more fragmented mitochondrial network than control cells, indicating a possible mitochondrial disfunction (Supplementary Fig. 4B). Further experiments should confirm these preliminary observations and deepen how ROS levels and mitochondrial function participate in the neurological syndrome caused by the HERC2 P594L variant.

## Discussion

**Fig. 8:**
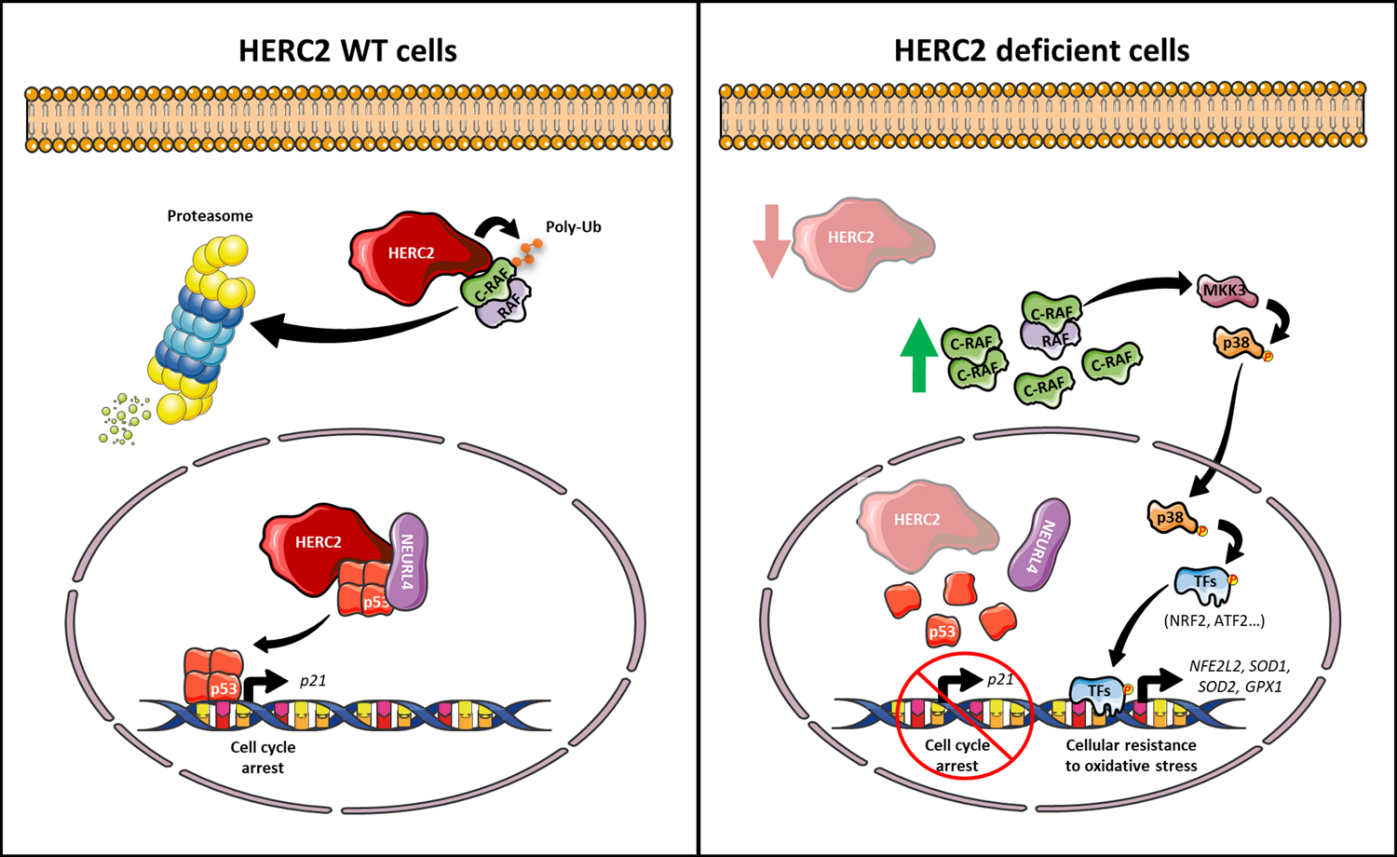
Working model of HERC2 function in health and disease. In previous studies, we showed that independently of the ubiquitin ligase activity, HERC2 along with NEURL4, facilitates p53 oligomerisation to promote p53 transcriptional program activation. For example, the target gene p21 regulates the cell cycle and promotes cell cycle arrest. Under conditions of HERC2 deficiency or down-regulation, the transcriptional activation of p53 is impaired due to the compromised p53 oligomerisation process [10–13]. Now, with data presented in this study, we complement this working model by adding an important function of HERC2 dependent on its ubiquitin ligase activity. Under normal conditions, HERC2 controls C-RAF protein levels by regulating its ubiquitylation and targeting it to proteasomal degradation. Hence, in HERC2-deficient cells, C-RAF protein levels increase, which activates a crosstalk between the C-RAF and MKK3/p38 signalling pathways. Once p38 is activated by phosphorylation, it translocates to the nucleus and activates its target transcription factors (TFs). This eventually activates transcription of genes related to the oxidative stress response such as *NFE2L2*, *SOD1*, *SOD2* and *GPX1*, which predisposes cells to an enhanced resistance to oxidative stress. The combination of these effects in the p53/p21 and MKK3/p38 pathways may affect both tumorigenesis and neuronal cell homeostasis

This study provides the first evidence that HERC2 controls the cellular response to oxidative stress through the p38 signalling pathway dependent on RAF. Our results demonstrate that HERC2 forms a complex with RAF proteins, consistent with the results of a previous proteomic analysis, in which C-RAF was identified to interact with the carboxyl-terminus domain of HERC2 [35]. Mechanistically, our data show that HERC2 regulates C-RAF ubiquitylation and protein degradation; thus, in individuals with the HERC2 P594L mutation, the resulting HERC2 deficiency, causes an increase in C-RAF protein levels. However, this increase is not signalled through the canonical MEK/ERK pathway, and instead, seems to affect the MKK3/p38 pathway specifically (Fig. 8). Activation of crosstalk between C-RAF and the MKK3/p38 pathway has also been described as a mechanism regulated by HERC1, the other member of the large HERC protein family [29]. This raises the question of whether this signalling mechanism is specific to large HERC proteins. In any case, our results demonstrated that the role of HERC2 in the C-RAF/MKK3/p38 signalling pathway is independent of HERC1. Several lines of evidences show this independent role: (1) HERC1 and HERC2 proteins do not interact [29]; (2) HERC1 is not present in the HERC2/C-RAF complex (Fig. 5A); and (3) while HERC1 depletion regulates ERK signalling, HERC2 does not (Fig. 2D). By contrast, activation dependent on HERC2 affects the cellular response to oxidative and saline stresses. Although the precise mechanism explaining the differences between HERC1 and HERC2 should be explored further, differences could be explained by the different complexes formed between RAF proteins and large HERC proteins or by the pleiotropy of the p38 pathway [36]. Many p38 MAPK substrates have been described, both in the cytosol and the nucleus, and each large HERC family member appears to direct p38 signalling towards different downstream targets, suggesting the participation of different HERC1 and HERC2 complexes.

NRF2, a transcription factor encoded by the *NFE2L2* gene, is considered the master regulator of the cellular antioxidant response [37]. A critical regulatory step leading to its activation is its dissociation from Cullin 3 (CUL3) and the ubiquitin ligase Kelch-like ECH-associated protein 1 (KEAP1). CUL3 ubiquitylates NRF2, targeting it to proteasomal degradation, and upon exposure to oxidative stress, the NRF2-KEAP1 complex is disrupted and NRF2 is stabilised for translocation to the nucleus. Nevertheless, the precise mechanism by which cellular stress signals end up reaching NRF2 and causing its dissociation of the complex remains unclear [38]. Indeed, several studies have pointed out that some MAPK pathways are responsible for regulating this signal transduction. The p38 MAPK can regulate NRF2 activity through its activation [39–41] and its repression [42] depending on the context [43]. We observed NRF2 protein levels increasing after HERC2 depletion (Fig. 6C), consistent with p38 activating NRF2. Moreover, the mRNA levels of NRF2-regulated antioxidant genes also increased (e.g. *SOD1*, *SOD2* and *GPX1*) (Fig. 6B). The *NFE2L2* gene contains an antioxidant response element within its promoter region, providing NRF2 the ability to activate its own transcription [44]. This could explain why we observed increased mRNA levels of *NFE2L2* in addition to its protein levels. In addition, the fact that the inhibition of RAF or p38 activity, abolished upregulation of NRF2 in HERC2-depleted cells, confirmed that p38 acts upstream of NRF2 activation. Still, given the variety of p38 substrates we cannot discard that other transcription factors, apart from NRF2, could also be involved in the regulation of the studied antioxidant genes. The transcription factor ATF-2 is another important mediator of p38 in the induction of *SOD2* expression upon H_2_O_2_-induced oxidative stress in MEFs [45]. This possible cooperation between NRF2 and ATF-2, or some other transcription factor targeted by p38, should be studied further.

Overall, our findings may have both physiological and clinical repercussions. Physiologically, we revealed a pro-survival function of p38 that is regulated by HERC2. HERC2 potentially fine-tunes the cellular response to oxidative stress by controlling protein levels of C-RAF and, therefore, C-RAF/MKK3/p38 signalling to regulate antioxidant gene expression. Clinically, these findings may be relevant to cancer, as well as individuals with HERC2 Angelman-like syndrome due to biallelic *HERC2* gene variants [16, 18].

Several *HERC2* mutations have been associated with a wide number of tumours [4]. In renal cancer, higher *HERC2* gene expression correlates with better patient prognosis [46], supporting the hypothesis that HERC2 may act as a tumour suppressor [4, 46]. We previously demonstrated that HERC2, and NEURL4, regulate the transcriptional activity of the tumour suppressor p53, facilitating its oligomerisation. HERC2 knockdown accordingly increases cell proliferation due to the impaired capability to arrest the cell cycle through p53 [10–12]. Equally, although the precise mechanism remains elusive, it is well established that the production of reactive oxygen species in tumour cells increases due to the higher metabolic rate, with the resulting excess being countered by an increased antioxidant cellular response [47]. Supporting this, in mice, oncogenic alleles of *Kras*, *Braf* and *Myc*, associated with increased *Nfe2l2* expression. This appear to stably enhance NRF2 antioxidant program and lower intracellular reactive oxygen species [48]. Furthermore, in p53 mutated cancer cells, the NRF2-dependent antioxidant response was selectively modulated to enhance cancer cell survival [49]. Our data reveal a new mechanism by which HERC2 deficiency may contribute to tumour malignancy by impairing p53 transcriptional activity, and also by boosting the cellular antioxidant response making cancer cells more resistant to oxidative stress (Fig. 8). In this context, combination treatments with drugs causing non-genotoxic activation of p53 oligomerisation and FDA-approved RAF inhibitors, such as Sorafenib, represent potential therapeutic candidates for tumours associated with HERC2 deficiency.

Finally, a previous proteomic analysis of human *HERC2* mutants (including the p.Pro594Leu variant studied here) has already identified an enrichment of the NRF2-mediated oxidative stress response in *HERC2* mutants compared to control group [20]. In addition, protein–protein interaction networks containing signal transduction proteins and MAPKs were found to be differentially expressed in *HERC2* mutants [20]. Our results add to these observations and, importantly, provide a possible molecular mechanism explanation. It would be interesting for further research to study the implication of a chronic activation of the C-RAF/MKK3/p38 signalling pathway in neuronal cells with HERC2 deficiency. Alterations in p38 MAPK signalling in neurons have been linked to neurodegenerative diseases including Parkinson’s disease, Alzheimer’s disease and amyotrophic lateral sclerosis (ALS) [50]. Therefore, we cannot discount the possibility that alterations in this pathway could be associated with clinical outcomes in HERC2 Angelman-like syndrome. Consistent with this, previous studies have shown that SOD1 overexpression, in which gene variants are associated with ALS and whose mRNA levels we found to be increased following HERC2 depletion, is associated with defects in the cerebellar architecture [51, 52]. In addition, while excessive ROS elicit oxidative stress, their persistent depletion, as observed in HERC2-deficient cells (Supplementary Fig. 4A), leads to an opposite condition called reductive stress. Persistent activation of antioxidant signalling can cause reductive stress and lead to pathology. In HERC2 P594L cells, the overactivation of NRF2 signalling could be one of the causes. In fact, NRF2 sustained activation has already been linked to reductive stress [53]. Highlighting the importance of reductive stress on pathology, mutations in key components of the cellular reductive stress response can cause developmental diseases. For instance, FEM1B gain-of-function mutation, which cause a persistent activation of the reductive stress response, elicit developmental syndromes with some similarities to the HERC2 Angelman-like syndrome [33, 34]. An example of the damage that reductive stress can exert on cells is that it can induce mitochondrial dysfunction and impact on the correct cell function [54, 55]. Accordingly, we observed an increased number of fragmented mitochondria in HERC2 P594L cells, which is a common feature observed in neurodegeneration [56]. However, more experiments are needed to confirm these hypotheses and to associate these mechanisms with clinical outcomes in HERC2 Angelman-like syndrome. All things considered, the findings in this study identify p38 and RAF inhibitors as potential therapeutic options for individuals who present with such rare disease.

## Supplementary Information

Below is the link to the electronic supplementary material.

Supplementary file1 (PDF 633 KB)

## Data Availability

All data analysed during this study to evaluate the conclusions are included within the article or available in supplemental information. Additional related data need to be requested from the corresponding author.

## References

[1] Parenti I, Rabaneda LG, Schoen H, Novarino G (2020). Neurodevelopmental disorders: from genetics to functional pathways. Trends Neurosci.

[2] García-Cano J, Martinez-Martinez A, Sala-Gaston J (2019). HERCing: structural and functional relevance of the large HERC ubiquitin ligases. Front Physiol.

[3] Husnjak K, Dikic I (2012). Ubiquitin-binding proteins: decoders of ubiquitin-mediated cellular functions. Annu Rev Biochem.

[4] Sala-Gaston J, Martinez-Martinez A, Pedrazza L (2020). Herc ubiquitin ligases in cancer. Cancers (Basel).

[5] Lee TH, Park JM, Leem SH, Kang TH (2014). Coordinated regulation of XPA stability by ATR and HERC2 during nucleotide excision repair. Oncogene.

[6] Peng Y, Dai H, Wang E (2015). TUSC4 functions as a tumor suppressor by regulating BRCA1 stability. Cancer Res.

[7] Yuan J, Luo K, Deng M (2014). HERC2-USP20 axis regulates DNA damage checkpoint through Claspin. Nucleic Acids Res.

[8] Zhu M, Zhao H, Liao J, Xu X (2014). HERC2/USP20 coordinates CHK1 activation by modulating CLASPIN stability. Nucleic Acids Res.

[9] Wu W, Rokutanda N, Takeuchi J (2018). HERC2 facilitates BLM and WRN helicase complex interaction with RPA to suppress G-quadruplex DNA. Cancer Res.

[10] Cubillos-Rojas M, Amair-Pinedo F, Peiró-Jordán R (2014). The E3 ubiquitin protein ligase HERC2 modulates the activity of tumor protein p53 by regulating its oligomerization. J Biol Chem.

[11] Cubillos-Rojas M, Schneider T, Bartrons R (2017). NEURL4 regulates the transcriptional activity of tumor suppressor protein p53 by modulating its oligomerization. Oncotarget.

[12] García-Cano J, Sánchez-Tena S, Sala-Gaston J (2020). Regulation of the MDM2-p53 pathway by the ubiquitin ligase HERC2. Mol Oncol.

[13] Cubillos-Rojas M, Schneider T, Hadjebi O (2016). The HERC2 ubiquitin ligase is essential for embryonic development and regulates motor coordination. Oncotarget.

[14] Pérez-Villegas EM, Ruiz R, Bachiller S (2021). The HERC proteins and the nervous system. Semin Cell Dev Biol.

[15] Puffenberger EG, Jinks RN, Wang H (2012). A homozygous missense mutation in HERC2 associated with global developmental delay and autism spectrum disorder. Hum Mutat.

[16] Harlalka GV, Baple EL, Cross H (2013). Mutation of HERC2 causes developmental delay with angelman-like features. J Med Genet.

[17] Morice-Picard F, Benard G, Rezvani HR (2016). Complete loss of function of the ubiquitin ligase HERC2 causes a severe neurodevelopmental phenotype. Eur J Hum Genet.

[18] Elpidorou M, Best S, Poulter JA (2021). Novel loss-of-function mutation in HERC2 is associated with severe developmental delay and paediatric lethality. J Med Genet.

[19] Vincent KM, Eaton A, Yassaee VR (2021). Delineating the expanding phenotype of HERC2-related disorders: the impact of biallelic loss of function versus missense variation. Clin Genet.

[20] Abraham JR, Barnard J, Wang H (2019). Proteomic investigations of human HERC2 mutants: Insights into the pathobiology of a neurodevelopmental disorder. Biochem Biophys Res Commun.

[21] Wu W, Sato K, Koike A (2010). HERC2 is an E3 ligase that targets BRCA1 for degradation. Cancer Res.

[22] Schneider T, Martinez-Martinez A, Cubillos-Rojas M (2018). The E3 ubiquitin ligase HERC1 controls the ERK signaling pathway targeting C-RAF for degradation. Oncotarget.

[23] Chan NC, Den Besten W, Sweredoski MJ (2014). Degradation of the deubiquitinating enzyme USP33 is mediated by p97 and the ubiquitin ligase HERC2. J Biol Chem.

[24] Erazo T, Moreno A, Ruiz-Babot G (2013). Canonical and kinase activity-independent mechanisms for extracellular signal-regulated kinase 5 (ERK5) nuclear translocation require dissociation of Hsp90 from the ERK5-Cdc37 complex. Mol Cell Biol.

[25] Ramirez J, Prieto G, Olazabal-Herrero A (2021). A proteomic approach for systematic mapping of substrates of human deubiquitinating enzymes. Int J Mol Sci.

[26] Moffat J, Grueneberg DA, Yang X (2006). A lentiviral RNAi library for human and mouse genes applied to an arrayed viral high-content screen. Cell.

[27] Casas-Terradellas E, Tato I, Bartrons R (2008). ERK and p38 pathways regulate amino acid signalling. Biochim Biophys Acta Mol Cell Res.

[28] Cubillos-Rojas M, Amair-Pinedo F, Tato I (2010). Simultaneous electrophoretic analysis of proteins of very high and low molecular mass using Tris-acetate polyacrylamide gels. Electrophoresis.

[29] Pedrazza L, Schneider T, Bartrons R (2020). The ubiquitin ligase HERC1 regulates cell migration via RAF-dependent regulation of MKK3/p38 signaling. Sci Rep.

[30] Raingeaud J, Gupta S, Rogers JS (1995). Pro-inflammatory cytokines and environmental stress cause p38 mitogen- activated protein kinase activation by dual phosphorylation on tyrosine and threonine. J Biol Chem.

[31] Brummer T, McInnes C (2020). RAF kinase dimerization: implications for drug discovery and clinical outcomes. Oncogene.

[32] Bellezza I, Giambanco I, Minelli A, Donato R (2018). Nrf2-Keap1 signaling in oxidative and reductive stress. BBA Mol Cell Res.

[33] Manford AG, Rodríguez-Pérez F, Shih KY (2020). A cellular mechanism to detect and alleviate reductive stress. Cell.

[34] Manford AG, Mena EL, Shih KY (2021). Structural basis and regulation of the reductive stress response. Cell.

[35] Galligan T, Martinez-noe G, Arndt V (2015). Proteomic analysis and identification of cellular interactors of the giant ubiquitin ligase HERC2. J Proteome Res.

[36] Canovas B, Nebreda AR (2021). Diversity and versatility of p38 kinase signalling in health and disease. Nat Rev Mol Cell Biol.

[37] Rojo de la Vega M, Chapman E, Zhang DD (2018). NRF2 and the Hallmarks of cancer. Cancer Cell.

[38] Suzuki T, Yamamoto M (2015). Molecular basis of the Keap1-Nrf2 system. Free Radic Biol Med.

[39] Kang KW, Ryu JIH, Kim SG (2000). The essential role of phosphatidylinositol 3-kinase and of p38 mitogen-activated protein kinase activation in the antioxidant response element-mediated rGSTA2 induction by decreased glutathione in H4IIE hepatoma cells. Mol Pharmacol.

[40] Alam J, Wicks C, Stewart D (2000). Mechanism of heme oxygenase-1 gene activation by cadmium in MCF-7 mammary epithelial cells: role of p38 kinase and Nrf2 transcription factor. J Biol Chem.

[41] Zipper LM, Mulcahy RT (2000). Inhibition of ERK and p38 MAP kinases inhibits binding of Nrf2 and induction of GCS genes. Biochem Biophys Res Commun.

[42] Yu R, Chen C, Mo YY (2000). Activation of mitogen-activated protein kinase pathways induces antioxidant response element-mediated gene expression via a Nrf2-dependent mechanism. J Biol Chem.

[43] Huang HC, Nguyen T, Pickett CB (2002). Phosphorylation of Nrf2 at Ser-40 by protein kinase C regulates antioxidant response element-mediated transcription. J Biol Chem.

[44] He F, Ru X, Wen T (2020). NRF2, a transcription factor for stress response and beyond. Int J Mol Sci.

[45] Gutiérrez-Uzquiza Á, Arechederra M, Bragado P (2012). p38α mediates cell survival in response to oxidative stress via induction of antioxidant genes: effect on the p70S6K pathway. J Biol Chem.

[46] Schneider T, Martinez-Martinez A, Cubillos-Rojas M (2019). Large HERCS function as tumor suppressors. Front Oncol.

[47] Perillo B, Di DM, Pezone A (2020). ROS in cancer therapy: the bright side of the moon. Exp Mol Med.

[48] Denicola GM, Karreth FA, Humpton TJ (2011). Oncogene-induced Nrf2 transcriptionpromotes ROS detoxification and tumorigenesis. Nature.

[49] Lisek K, Campaner E, Ciani Y (2018). Mutant p53 tunes the NRF2-dependent antioxidant response to support survival of cancer cells. Oncotraget.

[50] Correa SAL, Eales KL (2012). The role of p38 MAPK and its substrates in neuronal plasticity and neurodegenerative disease. J Signal Transduct.

[51] Jaarsma D, Haasdijk ED, Grashorn JAC (2000). Human Cu/Zn superoxide dismutase (SOD1) overexpression in mice causes mitochondrial vacuolization, axonal degeneration, and premature motoneuron death and accelerates motoneuron disease in mice expressing a familial amyotrophic lateral sclerosis mut. Neurobiol Dis.

[52] Afshar P, Ashtari N, Jiao X (2017). Overexpression of human SOD1 leads to discrete defects in the cerebellar architecture in the mouse. Front Neuroanat.

[53] Rajasekaran NS, Varadharaj S, Khanderao GD (2011). Sustained activation of nuclear erythroid 2-related factor 2/antioxidant response element signaling promotes reductive stress in the human mutant protein aggregation cardiomyopathy in mice. Antioxidants Redox Signal.

[54] Ma WX, Li CY, Tao R (2020). Reductive stress-induced mitochondrial dysfunction and cardiomyopathy. Oxid Med Cell Longev.

[55] Xiao W, Loscalzo J (2020). Metabolic responses to reductive stress. Antioxidants Redox Signal.

[56] Knott AB, Perkins G, Schwarzenbacher R, Bossy-Wetzel E (2008). Mitochondrial fragmentation in neurodegeneration. Nat Rev Neurosci.

