# Supplementary material for: HERC2 deficiency activates C-RAF/MKK3/p38 signalling pathway altering the cellular response to oxidative stress
Source: Cell Mol Life Sci. 2022 Oct 14;79(11):548. doi: 10.1007/s00018-022-04586-7 (PMC9568463; doi:10.1007/s00018-022-04586-7)
Supplement: Supplementary file 1 — Supplementary file1 (PDF 633 KB) [file 18_2022_4586_MOESM1_ESM.pdf]

Supplementary figure 1

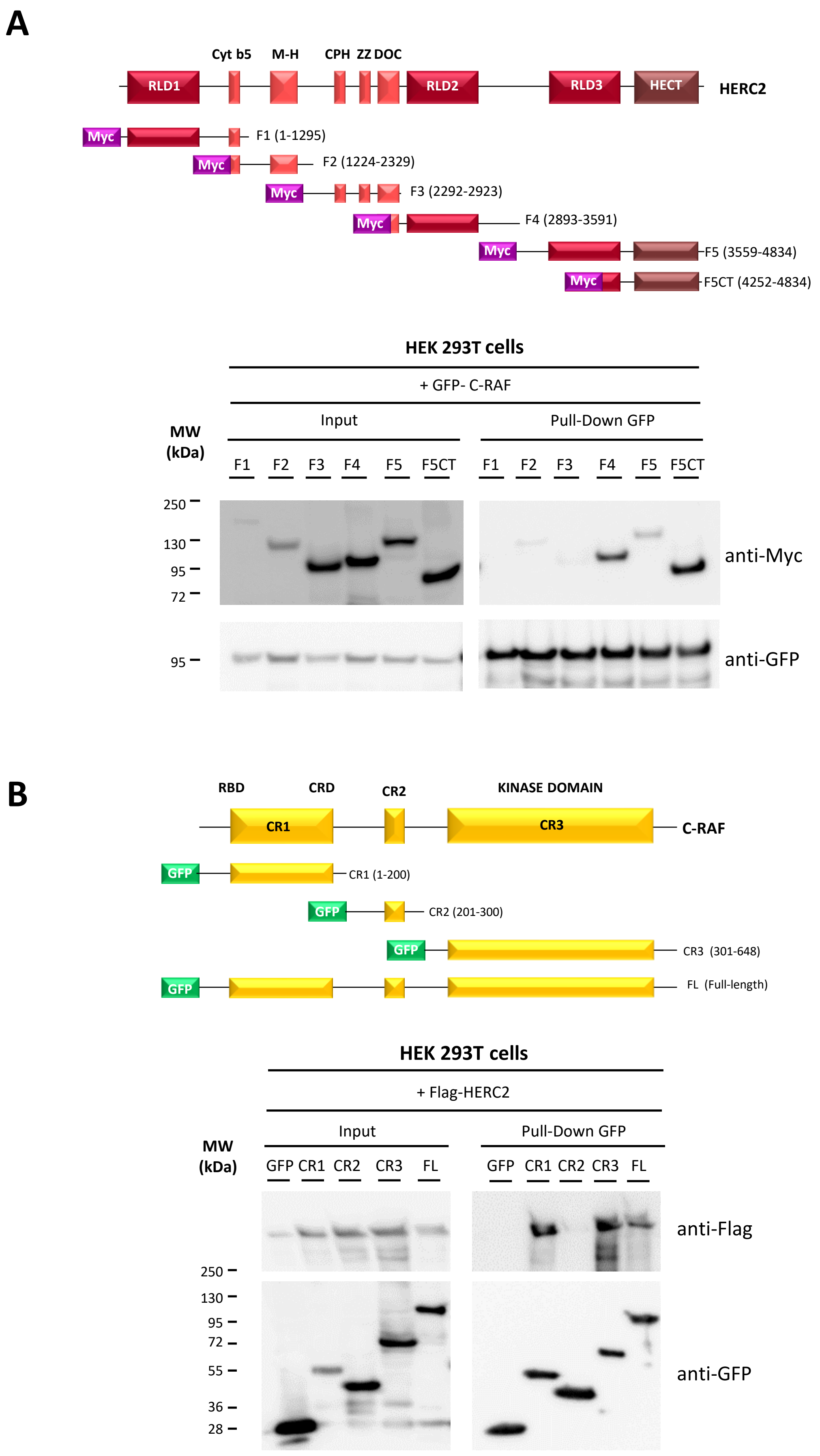

C

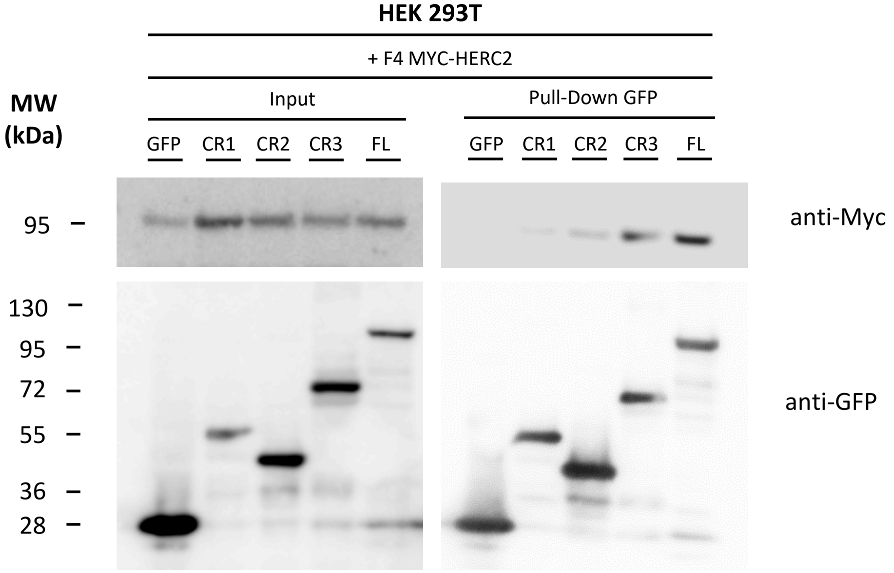

D

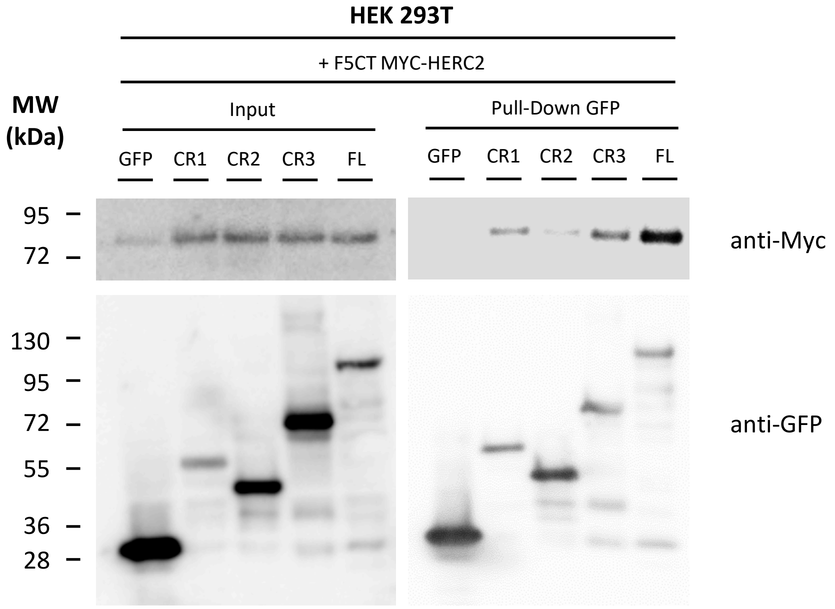

Supplementary figure 1. Domains involved in the interaction between HERC2 and C-RAF protein. (A) Schematic representation of HERC2 structure and its relevant domains is shown. The different Myc-HERC2 protein constructs are indicated. Pull-down experiments were performed in HEK 293T cells co-transfected with GFP-C-RAF and the indicated Myc-HERC2 fusion construct. 48 hours post-transfection, lysates from these cells were incubated with GFP-binding beads as indicated in “Materials and Methods”. Proteins retained in the resin were analyzed by immunoblotting with antibodies against the indicated proteins. **(B)** Schematic representation of C-RAF structure and its relevant domains is shown. The different GFP-C-RAF protein constructs are indicated. HEK 293T cells were co-transfected with full-length Flag-HERC2 fusion protein and the indicated GFP-C-RAF fusion construct or GFP as a negative control. A pull-down experiment was performed as in (A). **(C-D)** HEK 293T cells were co-transfected with F4 Myc-HERC2 (C) or F5CT Myc-HERC2 (D) fusion constructs and the indicated GFP-C-RAF fusion construct or GFP as a negative control. A pull-down experiment was performed as in (A). Representative results are shown from experiments repeated at least three times.

Supplementary figure 2

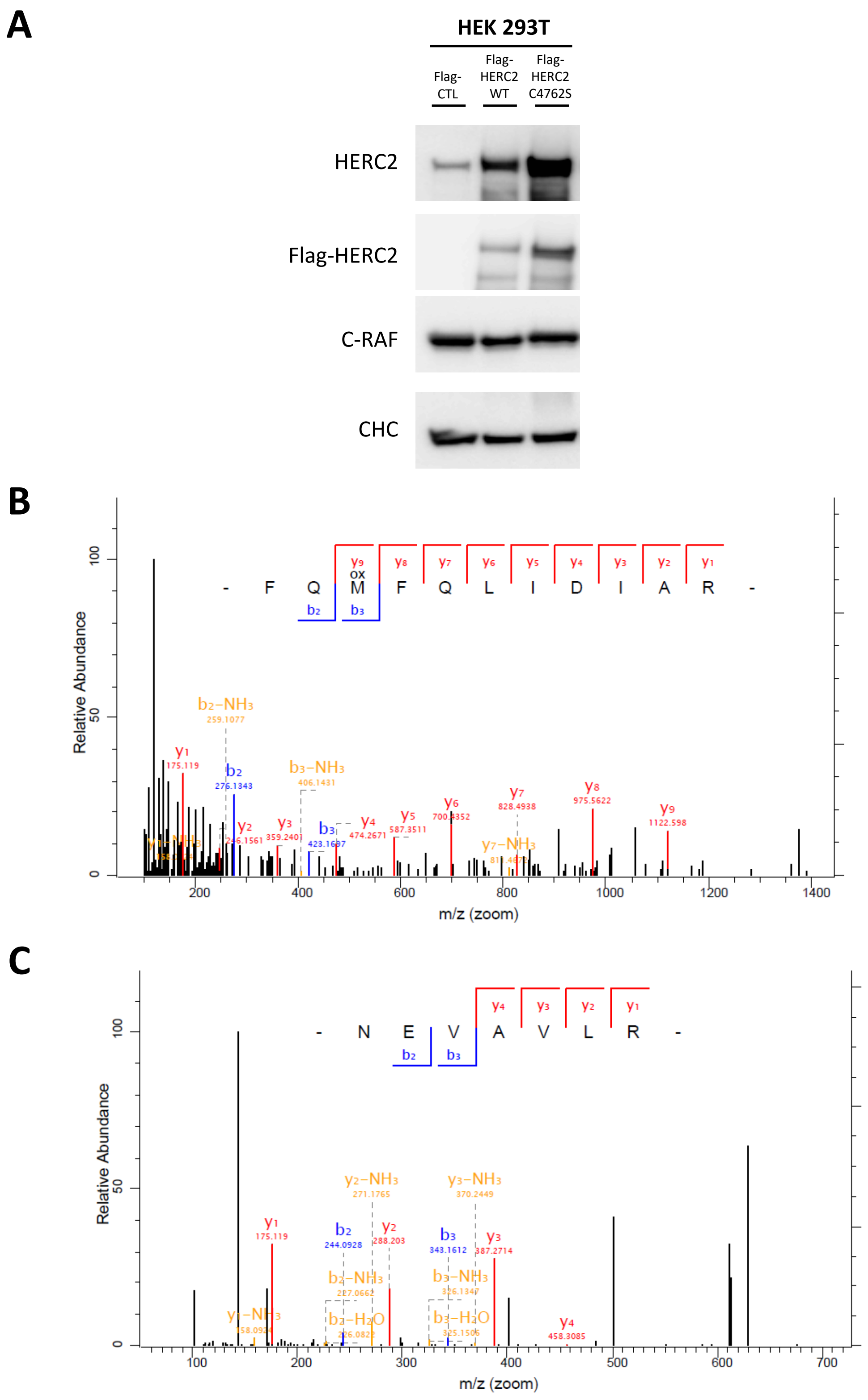

Supplementary figure 2. HERC2 regulates C-RAF ubiquitylation. (A) HEK 293T cells were transfected with a negative control plasmid (Flag-CTL), a plasmid encoding wild-type HERC2 protein (Flag-HERC2 WT) or a plasmid encoding a catalytically inactive form of HERC2 (Flag-HERC2 C4762S). The indicated proteins were analysed by immunoblot. Representative results are shown from experiments repeated at least three times. **(B-C)** Fragmentation spectrum of C-RAF peptides identified in ubiquitylated proteome from HEK 293T cells overexpressing Flag-HERC2 WT fusion protein by mass spectrometry, and not detected in control cells overexpressing Flag-HERC2 C4762S.

Supplementary figure 3

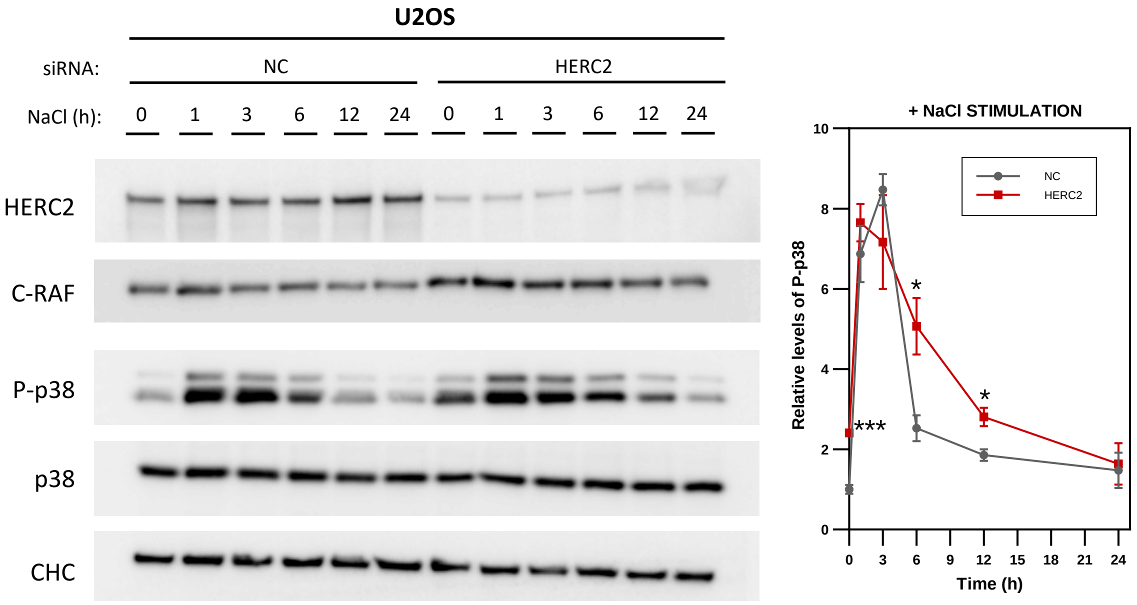

Supplementary figure 3. Regulation of the cellular stress response by HERC2. U2OS cells were transfected with an siRNA negative control (NC) or an siRNA against HERC2. Subsequently, cells were treated with 100 mM NaCl for the indicated time periods. The indicated protein levels were analyzed by immunoblot. Levels of phospho-p38 (P-p38) were quantified, normalized based on total p38 protein levels and expressed relative to the non-treated control condition (NC, t = 0). Plots represent mean ± standard error of the mean. Representative results are shown from experiments repeated 3 times (n = 3). Significance levels: ns = no significance * p ≤ 0.05; *** p ≤ 0.001.

Supplementary figure 4

A

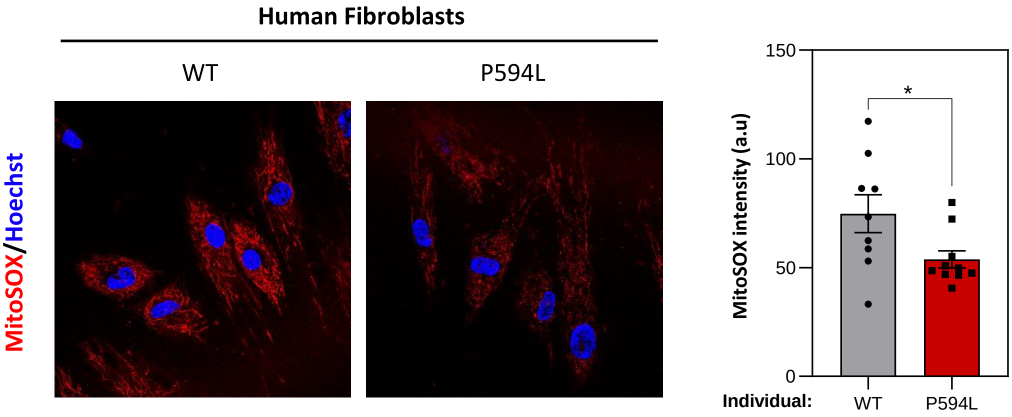

B

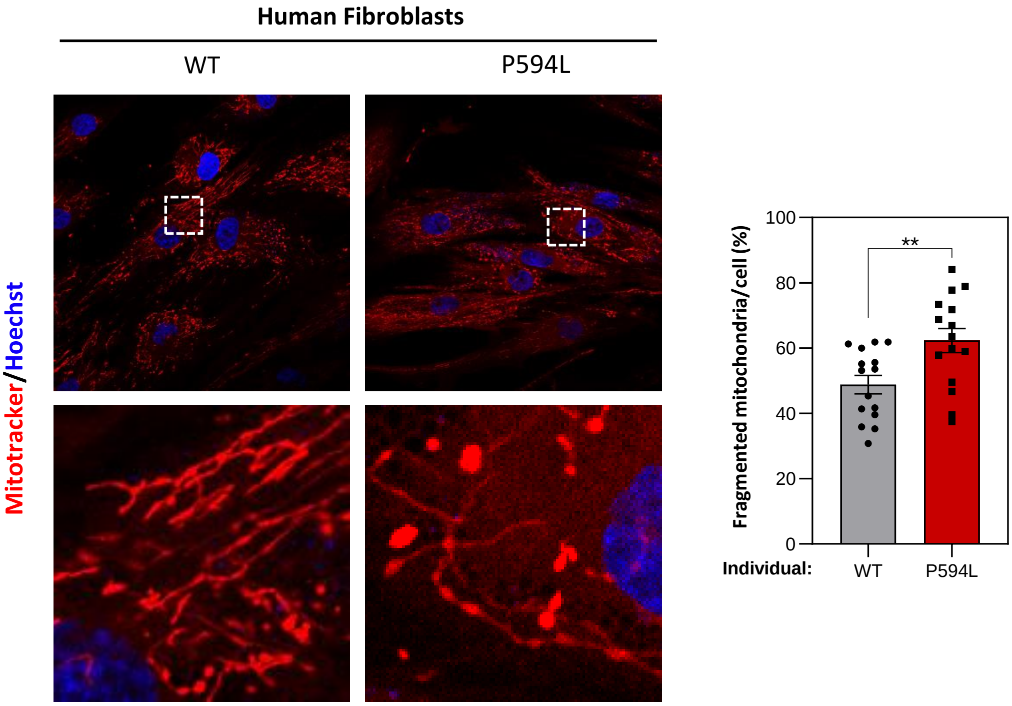

Supplementary figure 4. Presence of reductive stress in HERC2 P594L cells. (A) Human skin fibroblasts from an individual with the wild-type HERC2 (HERC2 WT) and the p.Pro594Leu mutant HERC2 variant (HERC2 P594L) were stained for mitochondrial superoxide using MitoSox (red). Nuclei were stained in blue with Hoechst. Red fluorescence intensity per cell was measured, quantified and expressed as arbitrary units (a.u). Each data point represent mean of a different field. **(B)** Mitochondrial morphology was examined in HERC2 WT and HERC2 P594L cells by immunofluorescence microscopy using Mitotracker staining (red). Nuclei were stained in blue with Hoechst. Fragmented mitochondrial percentage per cell was calculated. Each data point represent mean of a different field. Plots represent mean ± standard error of the mean. Representative results are shown from experiments repeated at least three times. Significance levels: ns = no significance * $p \leq 0.05$; ** $p \leq 0.01$.
